# A Green Approach to 2-Substituted Benzo- and Naphthothiazoles via *N*-bromosuccinimide/Bromide-Mediated C(aryl)-S Bond Formation

**DOI:** 10.3390/molecules27227876

**Published:** 2022-11-15

**Authors:** Ainka T. Brown, Nadale K. Downer-Riley

**Affiliations:** The Department of Chemistry, 2 Plymouth Crescent, The University of the West Indies, Mona, Kingston 7, Jamaica

**Keywords:** 2-aminobenzothiazole, benzothiazole, *N*-bromosuccinimide, naphthothiazole, oxidative cyclization, phenylthiourea

## Abstract

2-Substituted benzo- and naphthothiazoles have been conveniently prepared from the intramolecular cyclization of phenylthioureas and activated thiobenzanilides or the coupling of isothiocyanates with amines under mild conditions using *N*-bromosuccinimide/tetrabutylammonium bromide in 1,2-dimethoxyethane (DME) under ambient conditions. The reactions produce moderate to excellent yields with good functional group tolerance and avoid the use of harsh thermal conditions, corrosive reagents, halogenated solvents, toxic metal salts, and expensive metal catalysts, and are amenable to preparations on a gram-scale.

## 1. Introduction

**Scheme 1:**
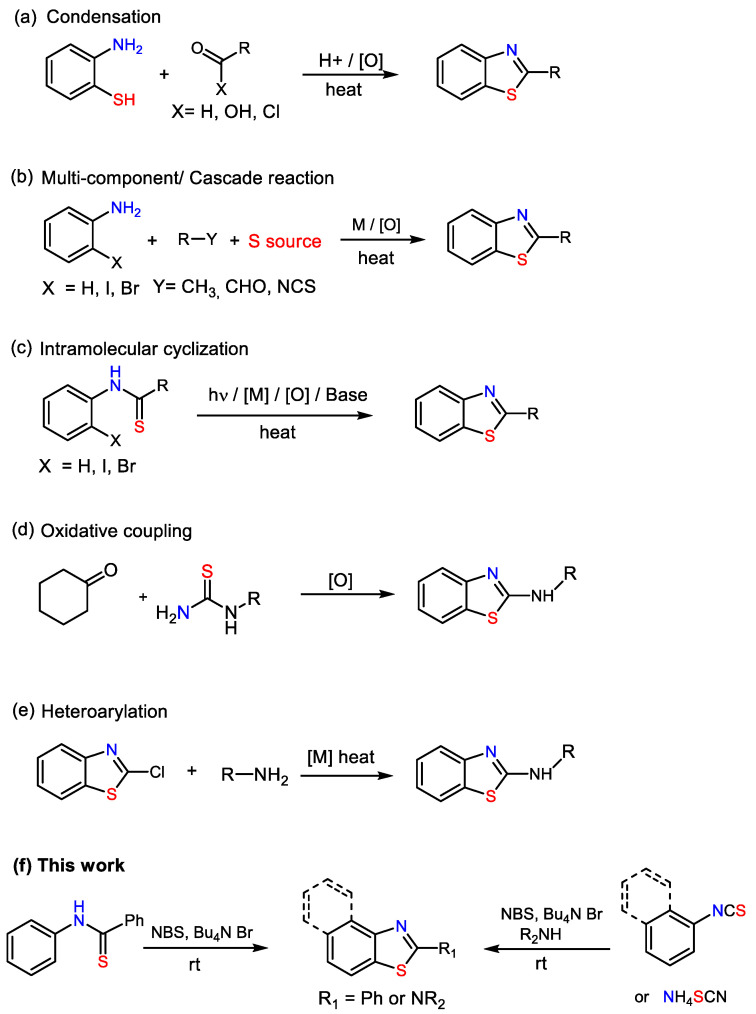
Strategies for benzothiazole synthesis.

The benzothiazole moiety is a feature in a wide array of naturally occurring and synthetic molecules with agricultural, pharmaceutical, and industrial applications. Compounds containing this structure possess myriad properties including fungicidal [1], anticancer [2], antimicrobial [3], anti-HIV [4], anti-convulsant [5], anti-inflammatory, and analgesic [6] activities, in addition to being used as imaging agents [7] and fluorescent dyes [8] among many other applications. Synthetic approaches to this heterocyclic core include the cyclocondensation of 2-aminothiophenols with carbonyl compounds and acid derivatives (Scheme 1, path a) [9,10], multicomponent reactions involving anilines or 2-iodoanilines with sulfur [11,12,13,14] (Scheme 1, path b), and the intramolecular cyclization of thioanilides (Scheme 1, path c) [15,16,17]. The oxidative cyclization of thioureas and cyclohexanones (Scheme 1, path d) [18], and the heteroarylation of amines with 2-halobenzothiazoles via both palladium-catalyzed and metal-free transformations, are additional approaches (Scheme 1, path e) [19,20]. There are some recent reviews in which the synthetic approaches towards the benzothiazole core are discussed in detail [21,22,23,24,25]. However, many of the methods utilized suffer from drawbacks such as a limited availability of appropriately functionalized precursors, functional group incompatibility, and the need for elevated temperatures, corrosive and toxic reagents, and metal catalysts including Cu(I), Cu(II), Ni(II), Pd(II), Sn(II), Fe(III), Ru(III), Fe(III), and Ti(IV) salts. Additionally, other methods also require the use of halogenated solvents with potential carcinogenicity and ozone-depleting potential. Therefore, greener strategies that minimize or avoid these undesirable environmental, health, and safety challenges and/or have lowered energy requirements are needed. Thus, efforts continue towards the development of milder, greener, and more cost-effective approaches for the construction of a benzothiazole core from readily available precursors.

The *N*-halosuccinimides are readily accessible, inexpensive, and easily handled crystalline compounds that are widely used in electrophilic halogenation reactions and as mild oxidants [26]. While other halogenating agents such as elemental bromine, chlorine, iodine [27], organic ammonium tribromides (OATBs) [28], and hypervalent iodine reagents [29,30] have frequently been employed to prepare 2-substituted benzothiazoles from thioureas and thiobenzanilides, there are far fewer instances of the use of *N*-halosuccinimides in these types of oxidative cyclization reactions. Giles [31] and Melendez and their co-workers [32] have indicated that treating phenylthioureas with *N*-bromosuccinimide (NBS) under strongly acidic conditions (MsOH/AcOH and concentrated sulfuric acid, respectively) produced 2-aminobenzothiazoles at a good yield. Moghaddam and Zargarani [33] have reported the use of NBS and milder conditions (CH_2_Cl_2_/CCl_4_ and room temperature) in the preparation of 2-arylbenzothiazoles from thiobenzanilides and Jickhar and co-workers have used *N*-iodosuccinimide (NIS) in CH_2_Cl_2_ to prepare 2-aminobenzothiazoles [34], while Chakabarty et al. [35] was able to effect the preparation of a tricyclic thiazolo[5,4-*e*]indole core using NBS with a strong base at low temperatures (CH_2_Cl_2_, −10 °C). To the best of our knowledge, there are no other reports of *N*-halosuccinimides being used in this manner.

In line with the ongoing efforts to prepare structurally and biologically interesting molecules containing 1,3-benzothiazole and bis-benzathiazole cores [27,30,36], we were interested in the utility of these halogen transfer agents to prepare 2-substituted benzothiazoles from their thiourea or thiobenzanilide precursors. Herein, we report a green approach for the preparation of 2-substituted benzothiazoles using *N*-halosuccinimides and quaternary ammonium halides in 1,2-dimethoxyethane (DME) under mild conditions via stepwise and one-pot approaches. This method does not require the use of heat, a metal catalyst, or corrosive reagents, and unlike previous reports that utilized *N*-halosuccinimides [33,34,35] a halogenated solvent was not used. Therefore, this method presents a more environmentally benign and greener approach.

## 2. Results and Discussion

The halogen-mediated synthesis of 2-substituted benzothiazoles from phenylthioureas and thiobenzanilides is thought to occur via the halogenation of the thiocarbonyl sulfur followed by an intramolecular electrophilic aromatic substitution [27,28]. It was expected that the use of *N*-halosuccinimides would allow for product formation in a similar manner. Phenylthiourea (**1**) was chosen as the model substrate and was treated with one equivalent of NBS in various solvents at 0 °C and at ambient temperature (Table 1).

The oxidative cyclization producing benzothiazole **2** occurred in low to fair yields (3–48%). The best yields were observed with halogenated and/or moderately polar solvents while highly polar solvents such as AcOH, MeCN, MeOH, EtOH, water and DMSO produced significantly lower yields of **2** (3–23%; entries 5–10), presumably due to the competing ring bromination reaction and/or solubility issues.

Despite some of the halogenated solvents giving superior yields, we opted to further explore the use of the more environmentally benign solvents. Reactions using several of the non-halogenated solvents were repeated at reflux but disappointingly resulted in more complex mixtures (entries 4–9). Of the solvents tested, DME was selected for further optimization and the effects of reduced temperature and the choice of *N*-bromosuccinimide (NBS), *N*-chlorosuccinimide (NCS), or *N*-iodosuccinimide (NIS) were also explored. Lowering the temperature resulted in a decrease in product formation to 25% (entry 4), while the performance of NBS and NIS at room temperature were comparable (40%) and slightly superior to that of NCS (33%) (Table 1).

The outcome of the reactions in which the *N*-halosuccinimides (NXS) provide a source of halonium ions (X^+^) has been shown to be influenced by the addition of a source of halide ions (X^−^) [37]. We decided to also examine how this would affect benzothiazole formation by having a solution of compound **1** and one equivalent of quaternary ammonium halide stirred together for 5 min prior to the introduction, in portions, of one equivalent of the corresponding NXS (Table 1, entries 13–16). There was a significant improvement in the yield upon combining NBS with a source of Br^−^ ions (entry 13), in which product formation increased from 40% to 60%. The NCS/Cl^−^ and NIS/I^−^ combinations, however, led to inferior results, with moderate and significant reductions in yield and gave only 27% of the product in the case of the former and no benzothiazole **2** in the case of the latter (entries 15 and 16). As such, NBS was chosen as our preferred oxidant.

A plausible explanation for these results is the formation of an NXS/X^−^ complex [37] that is in equilibrium with the succinimide anion and corresponding halogen, in which the latter acts as the effective oxidant. The yields obtained parallel the oxidizing ability of the respective halogens and the ability of the likely sulfenyl halide intermediate to facilitate cyclization onto the aromatic ring. The screening of other bromide sources such as HBr and LiBr (entries 17 and 18) indicated that tetrabutylammonium bromide (Bu_4_NBr) was optimal and the use of acetic acid (entry 14) as a solvent did not afford superior results in comparison to DME. To further our efforts towards green synthesis, ethanol was used as a solvent, with a poorer reaction outcome (28%; entry 19). Our screening results highlighted that increasing the polarity of the solvent favored competing bromination reactions as did an increase in temperature. Lower temperatures impeded the conversion of the starting material.

With the optimized conditions determined (Table 1, entry 13), we explored the reaction scope and limitations of our protocol using a series of substituted phenylthioureas and thiobenzanilides. The effects of electron-donating (CH_3_, OCH_3_) and electron-withdrawing (Br, NO_2_) aromatic substituents as well as alkyl and aryl nitrogen substituents were assessed (Table 2).

**Figure 1:**
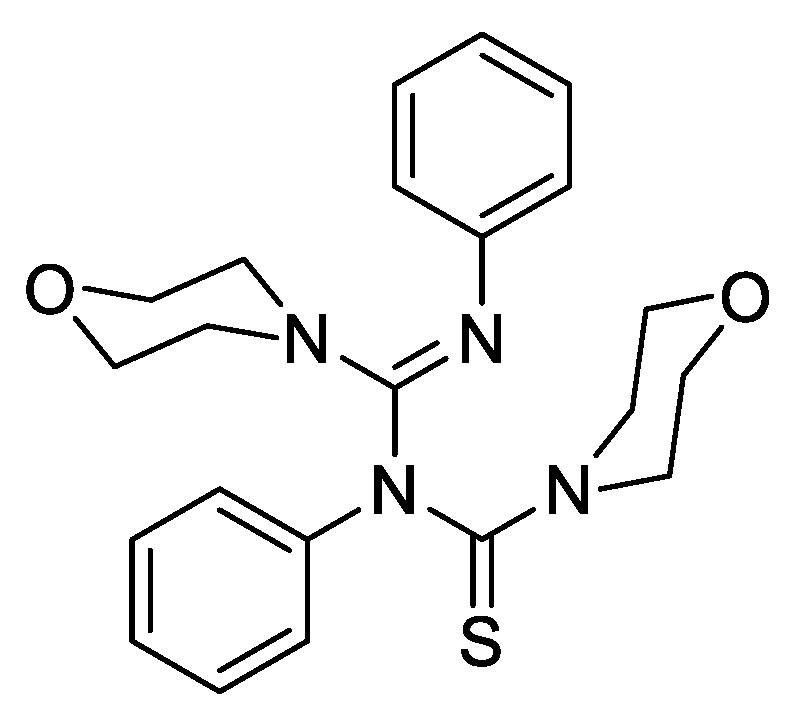
Thioamidoguanidine **7**, an “anti-Hugerschoff” product.

In line with our expectations, in the presence of NBS/Bu_4_NBr, the electron-rich aryl rings of substrates **3a**,**b** facilitated cyclization to produce benzothiazole **5** at 39 and 50% yields (Table 2). However, this was also accompanied by the formation of small quantities of a ring-brominated by-product. The use of the less reactive NCS/Me_4_NCl as an oxidant for these compounds avoided the competing ring halogenation and solely produced the desired benzothiazoles **5a** and **5b** in yields of 27% and 47% (Table 2). Despite the presence of the moderately electron withdrawing 4-bromo substituent on phenylthiourea **3c**, its aromatic ring was sufficiently nucleophilic for benzothiazole formation to proceed at a 53% yield. However, compound **3d**, which has a strongly electron withdrawing nitro group at the 4-position, produced no product, indicating that the electron-poor aromatic ring was unable to participate in the necessary C_(aryl)_-S bond formation under these conditions. Phenylthioureas **3e**–**g** with a monosubstituted nitrogen cyclized efficiently (56–84% yield) to produce the corresponding 2-aminobenzothiazoles **5e**–**g**. *N*,*N*-Disubstituted phenylthiourea **3h,** however, did not produce the expected product **5h** (Table 2) but instead the ‘anti-Hugerschoff’ product, compound **7** (Figure 1), at a 70% yield with spectroscopic data showing the presence of the thiocarbonyl (185 ppm; 1633 cm^−1^) and imino (149 ppm; 1586 cm^−1^) moieties. This is in line with reports suggesting that under various oxidizing conditions, aryl *sec*-alkyl thioureas can preferentially undergo dimerization and rearrangement to generate a thioamidoguanidine rather than undergoing cyclization to produce a benzothiazole [38].

We also explored the use of our protocol to prepare 2-phenylbenzothiazoles from thiobenzanilides. The use of NBS/Bu_4_NBr was less successful in this regard as cyclization only occurred in the presence of activated rings and with low to moderate yields of 5–57% (Table 2). Unlike the report by Moghaddam and Zargarani [33], in our experiment, thiobenzanilide (**4a**) failed to produce 2-phenylbenzothiazole (**6a**). Irrespective of the *N*-halosuccinimide used, the presence of halide anions, or solvent polarity, the rapid desulfurization of **4a** occurred and produced the corresponding benzanilide or both desulfurization and ring halogenation occurred when the reaction was conducted at high temperatures (see Supplementary Materials). The electron-releasing CH_3_ and OCH_3_ functional groups in substrates **4b**–**f** slightly improved reaction outcomes, producing small quantities of product in which the best yields were obtained with the dimethoxy-substituted **6e** and the 3-methoxy-substituted thiobenzanilide **6f** (Table 2). However, in all cases, cyclization was accompanied by desulfurization. Our results suggest that for thioanilides **4a**–**f**, while halogenation of the thiocarbonyl sulfur readily occurs, the intermediate formed is insufficiently electrophilic for appreciable benzothiazole formation and preferentially undergoes the expulsion of sulfur to produce an amide.

The direct (one-pot) synthesis of 2-aminobenzothiazoles from anilines and (iso)thiocyanates was also examined (Table 3). It was anticipated that the phenylthioureas formed in situ via the reaction of amines with (iso)thiocyanate would, in the presence of our brominating mixture, readily proceed to form 2-aminobenzothiazole. The treatment of a mixture of *p*-toluidine (**7a**), ammonium thiocyanate, and Bu_4_NBr in DME with NBS at ambient temperature yielded benzothiazole **5a** at a 51% yield while the reaction with *p*-anisidine (**7b**) gave product **5b** at a 54% yield. The less nucleophilic *p*-bromoaniline (**7c**), however, only afforded 20% of the corresponding benzothiazole **5c**. As anticipated, the coupling of *p*-toluidine (**7a**) with ethylisothiocyanate (**8a**) yielded the expected benzothiazole product **5i** at a good yield (Table 3).

The coupling of phenylisothiocyanate (**8b**) with substituted anilines, benzylamine, and primary aliphatic amines using a one-pot approach was also successful (Table 3). Stirring a solution of compound **8b**, Bu_4_NBr, and amino compounds for 45 min to 2 h (to generate the thiourea) with a subsequent introduction of NBS gratifyingly facilitated cyclization to produce the desired aminobenzothiazoles **5e**–**g** and **5j**–**n** in fair yields (42–60%). However, the reaction of compound **8b** and the secondary amine morpholine yielded none of the desired compound but instead provided thioamidoguanidine **7** at an excellent yield (90%). While switching the solvent to acetic acid and initiating the reaction at a lower temperature (10 °C) resulted in moderate to slight improvements when using anilines **7a**–**c** (Table 3), no conversion occurred under those conditions for the treatment of phenylisothiocyanate (**8b**) with benzylamine or aniline (Table 3). *N*-Bis-benzothiazoles are a class of bioactive benzothiazole-derived compounds that exhibit antiproliferative activity [39], while compounds with the phenyl benzothiazolyl urea functionality are well known to have wide-ranging bioactivity including antiviral, immunosuppressive, antithrombotic, and β-amyloid inhibitory properties [40,41]. A clinically relevant example of such an aryl urea is frentizole, which is used as a treatment for arthritis and systemic lupus among other conditions. As such, we attempted to extend our one-pot procedure to the preparation of bis-benzothiazole **5o** and urea **5p** from phenylisothiocyanate (**8b**) using phenylurea and 2-aminobenzothiazole **5a** as nucleophiles, respectively. However, their poor ability to act as nitrogen nucleophiles under the conditions resulted in only the starting material being observed. This remained the case even when the reaction time was extended and the temperature increased.

The scope of the substrates was successfully expanded by the replacement of phenylisothiocyante (**8b**) with naphthylisothiocyanate (**8c**), and, in general, excellent yields (83–95%) of naphthothiazoles were observed (Table 3). It was noted that the reactions towards producing naphthothiazoles **5r** and **5u**—in which an ammonium salt was combined with carbonate, as opposed to the use of a free amine—were less efficient (30% and 47%). We had anticipated that despite the limited solubility of the salts in DME there would be a gradual release of ammonia and dimethylamine, which would then proceed to react with thiocyanate **8c** and yield the thiourea intermediates. However, in both instances the conversion of **8c** remained incomplete, resulting in lower yields of the thiazole products.

Unlike phenylisothiocyanate (**8b**), naphthylisothiocyanate (**8c**) performed equally well with both primary and secondary amines. The reaction of morpholine with **8c** provided the desired naphthothiazole product at an 85% yield, which contrasts with the phenylisothiocyanate (**8b**) and morpholine reactions in which the dominant pathway is the formation of the ‘anti-Hugerschoff’ product. A similar outcome was seen for the reaction of naphthylisothiocyanate (**8c**) with pyrrolidine, which allowed for ready access to thiazole **5w** at a yield of 83%. Naphthothiazoles are far less represented in the literature compared to benzothiazoles. However, the examples reported have been used in biochemical screenings and for exploring enzyme functions and activity [42]; thus, it is useful to increase the number of tools available for their preparation. The improved yields of thiazole in the reactions using isothiocyanate **8a** and **8c** compared to **8b**, and the suppression of the thioamidoguanidine side-reaction when using the more sterically demanding secondary amines, are likely the result of more electron-rich aromatic rings that can better facilitate cyclization and C_(Ar)_-S bond formation under our conditions.

To date, the main approaches towards obtaining thiazoles **5a**–**5w** starting from (iso)thiocyanate have required one or more of the following: the use of liquid bromine, reactions that are conducted in a halogenated solvent [12,28,34,42,43,44], an oxygen atmosphere, elevated temperatures, the addition of a co-oxidant or a metal catalyst [13,14], or the pre-introduction of a halide leaving group [13]. In comparison, we consider our protocol to be a synthetically useful and operationally facile alternative that avoids these requirements and allows for the use of simple starting materials.

**Scheme 2:**
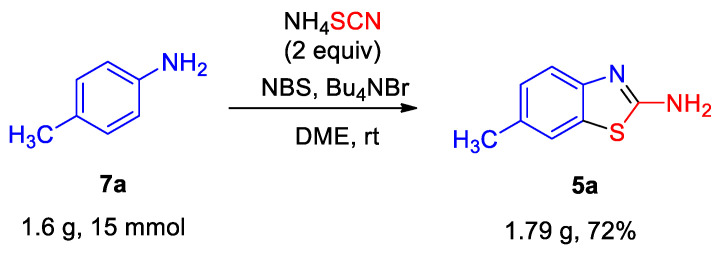
Gram scale synthesis of **4a**.

Next, we extended our study to explore the suitability of our one-pot protocol for benzothiazole synthesis on a large scale with *p*-toluidine (**7a**) used as a representative example (Scheme 2). A solution of 15 mmol of **7a** in DME was treated with 30 mmol of ammonium thiocyanate with NBS/Bu_4_NBr used as an oxidant. After 24 h, the target compound **5a** was obtained in gram quantities representing a yield of 72% and indicating that the reaction was more efficient on a large scale.

**Scheme 3:**
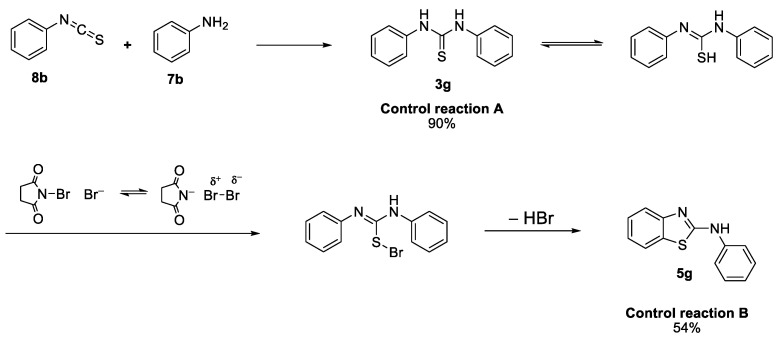
Control studies and proposed mechanism.

Taking our observations into consideration, as well as the literature reports on the use of halogen oxidants to prepare benzothiazoles, we propose that our reaction is most likely initiated by the formation of the sulfenyl bromide of the corresponding thiourea, which was added directly or formed in situ from phenylisothiocyanate (**8b**). The sulfenyl bromide can then undergo nucleophilic attack by the pi-electrons of the aryl ring with subsequent re-aromatization to produce the desired product. Control experiments in which we explored the likely intermediate(s) formed and the potential for a homolytic or a heterolytic reaction pathway were conducted to gain further insight (Scheme 3). Diphenylthiourea (**3g**) was obtained at a 90% yield by stirring a mixture of phenylisothiocyanate (**8b**), aniline, and Bu_4_NBr in DME (Scheme 3, control reaction A). This indicates that the thioureas are generated under our conditions, that this precedes product formation, and that NBS is required for cyclization to occur. Reactions were also carried out using our standard reagents and solvent, to which two molar equivalents of the radical inhibitor butylated-hydroxy toluene (BHT) were added. The presence of BHT had no noticeable effect, with the expected benzothiazole **5g** being obtained with no significant change in yield. These results suggest an ionic as opposed to radical pathway (Scheme 3).

Reaction conditions: Phenylisothiocyanate **8b** (1 mmol) and Aniline **7b** (1 molar equiv.); Control reaction A—Bu_4_NBr (1 molar equiv.), DME (4 mL), and rt (produces **3g**, 90%); Control reaction B—Bu_4_NBr (1 molar equiv.), NBS (1 molar equiv.), BHT (2 molar equiv.), DME (4 mL), and rt (produces **5g**, 54%)

## 3. Materials and Methods

### 3.1. General Information

Reagents and solvents were obtained from commercial sources (Sigma-Aldrich, St. Louis, MO, USA) and used as received without further purification. Unless otherwise stated, reactions were carried out under an air atmosphere. AcOH refers to glacial acetic acid; CH_2_Cl_2_ refers to methylene chloride; DME refers to 1,2-dimethoxyethane; EtOAc refers to ethyl acetate; EtOH refers to ethanol; and NMP refers to *N*-methyl-2-pyrrolidone. Thin-layer chromatography (TLC) was carried out using silica gel pre-coated alumina plates (200 µm) and visualized using ultraviolet light. Column chromatography was performed using silica gel (200–400 mesh).

^1^H NMR and ^13^C NMR spectra were recorded in CDCl_3_, acetone-d_6_, or DMSO-d_6_ on Bruker Avance 200 and 500 MHz spectrometers (Bruker Corporation, Delaware, USA). Chemical shifts are reported in parts per million (δ). Coupling constants (*J*) are reported in hertz (Hz). The following abbreviations for multiplicities are used: s = singlet, br s = broad singlet, d = doublet, t = triplet, q = quartet, and m = multiplet.

Melting points were recorded using a Gallenkamp melting point apparatus (Gallenkamp, Cambridge, UK) and open capillary tubes (Table 4). IR spectra were obtained using a Bruker Vector 22 spectrometer (Bruker Corporation, Delaware, USA).). Known compounds were identified by comparing their spectroscopic data and physical properties with those in the literature [12,13,14,15,18,27,28,30,38,43,45,46,47,48,49,50,51,52,53,54,55,56,57]. The characterization data of synthesized compounds are given in the Supplementary Materials.

### 3.2. General Procedures for Preparation of Substrates

#### 3.2.1. Method for Preparation of Phenylthioureas

Known procedures were used to prepare phenylthioureas **1**, **3a**–**3d** [44] (Method A), and **3e**–**3h**, [45,46] (Method B) from anilines and/or amines.

#### 3.2.2. Method for Preparation of Thiobenzanilides

The modification of a known procedure [47,48] (Method C) was used to prepare thiobenzanilides **4a**–**4c** from benzaldehydes and anilines.

Potassium carbonate (2 molar equiv.) and sulfur (3 molar equiv.) were thoroughly combined in a Smith process vial. NMP (3 mL/mmol of aniline) was introduced followed by the aniline (1 molar equiv.) and the suspension was vigorously stirred at room temperature until it became blue. Benzaldehyde (1.5 molar equiv.) was then added, and the vial was sealed with a Teflon cap and aluminum crimp. The reaction mixture was heated—with vigorous stirring applied—at 110 °C for 24 h. Upon cooling to room temperature, the mixture was carefully poured onto crushed ice (100–200 g) and left to stand until the product precipitated. Collection by suction filtration and purification by silica gel column chromatography using hexane–EtOAc as an eluent, yielded the products in the form of yellow solids.

A known method [27] (Method D) was used to prepare thiobenzanilides **4d**–**4f** from benzanilides.

### 3.3. General Procedures for Preparation of Benzothiazoles

#### 3.3.1. Intramolecular Cyclization of Phenylthioureas and Thiobenzanilides (Method E)

The phenylthiourea or thiobenzanilide substrate was either dissolved to produce a 0.25 M solution or suspended in DME. Quaternary ammonium halide (1 molar equiv.) was then added. Care was taken to exclude moisture. After stirring for approximately 5 min, *N*-halosuccinmide (1 molar equiv.) was added in portions. The reaction was vigorously stirred at ambient temperature until TLC indicated the consumption of starting material (4–24 h), following which it was slowly poured onto crushed ice (50 g). Concentrated aqueous ammonia was then used to adjust the pH to ~10. The precipitated solids were collected and dried by suction filtration. If precipitation did not occur, then the aqueous phase was extracted with EtOAc (3 × 15 mL), and the organic phase was washed with brine (15 mL), dried over magnesium sulfate, and concentrated under reduced pressure. Purification of the crude material was performed using column chromatography with hexane–EtOAc or hexane−CH_2_Cl_2_ as an eluent.

#### 3.3.2. One-Pot Synthesis Using Anilines and Ammonium Thiocyanate (Method F)

##### Using DME as Solvent

A solution of the aniline (3 mmol), ammonium thiocyanate (2 molar equiv.), and Bu_4_NBr (1 molar equiv.) in DME (5 mL) was stirred at room temperature for 5 min. Care was taken to exclude moisture. *N*-bromosuccinimide (1 molar equiv.) was then added in portions. After stirring vigorously at ambient temperature until TLC indicated the consumption of starting material (5–24 h), the mixture was poured onto crushed ice (50 g) and made basic (pH ~ 10) using concentrated aqueous ammonia. The precipitated solids were collected and dried by suction filtration. If precipitation did not occur readily, then extraction with EtOAc (3 × 20 mL) was carried out. The organic phase was washed with brine (20 mL), dried over magnesium sulfate, and concentrated under reduced pressure. Purification of the crude material was performed using column chromatography with hexane–EtOAc as eluent.

##### Using Glacial Acetic Acid as Solvent

A solution of aniline (3 mmol) and ammonium thiocyanate (12 mmol) in glacial AcOH (10 mL) was stirred at ambient temperature for 10 min. Care was taken to exclude moisture. The mixture was cooled to 10 °C and Bu_4_NBr (3 mmol) was introduced to produce a suspension. *N*-bromosuccinimide (3 mmol) was then added in portions over 10 min whilst still cooling. The reaction was allowed to gradually warm to ambient temperature, at which it was stirred for 24 h. The mixture was poured into an ice and water mixture (~150 mL) and the pH was adjusted to ~10 using concentrated aqueous ammonia. The precipitated solids were collected and dried by suction filtration. If precipitation did not occur readily, then extraction with EtOAc (3 × 20 mL) was carried out. The organic phase was washed with brine (20 mL), dried over magnesium sulfate, and concentrated under reduced pressure. Purification of the crude material was performed using column chromatography with hexane–EtOAc as eluent.

#### 3.3.3. One-Pot Synthesis Using Isothiocyanate and Amines (Method G)

A solution of isothiocyanate (1 mmol) and Bu_4_NBr (1 mmol) in DME (4 mL) was stirred at ambient temperature for 5 min. Care was taken to exclude moisture. Amine (1.1 mmol) was then added slowly, and the reaction mixture was allowed to stir until isothiocyanate was consumed as determined by TLC (45 min to 2 h). *N*-bromosuccinimide (1 mmol) was then added in portions and the mixture allowed to stir vigorously at ambient temperature. After TLC indicated the consumption of the intermediate compound and the formation of product after 1–20 h, the mixture was poured onto crushed ice (50 g) and made basic (pH ~ 10) using concentrated aqueous ammonia. The precipitated solids were collected and dried by suction filtration. If precipitation did not occur readily, then the aqueous phase was extracted with EtOAc (3 × 15 mL). The organic phase was washed with brine (15 mL), dried over magnesium sulfate, and concentrated under reduced pressure. Purification of the crude material was performed using either recrystallization (EtOH-H_2_O) or column chromatography with hexane–EtOAc as eluent.

## 4. Conclusions

A mild and convenient method for the synthesis of 2-substituted benzo- and naphthothiazoles from thioureas, activated thiobenzanilides, or amines and isothiocyanates, using *N*-bromosuccimide and tetrabutylammonium bromide, has been developed. The synthetically useful features of this protocol include the use of inexpensive and easily handled reagents as oxidants—thereby eliminating the need for metal catalysts, harsh conditions, and toxic or environmentally unfriendly reagents—and the provision of a method that can be conveniently carried out under an air atmosphere and at ambient temperatures. The method also allows for the gram-scale, one-pot formation of 2-aminobenzothiazoles from commercially available anilines.

## Figures and Tables

**Table 1:** Effect of solvent, *N*−halosuccinimde, and halide source on 2-aminobenzothiazole formation ^a^.

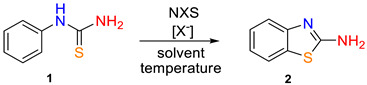				
Entry	NXS	[X^−^]	Solvent	Yield (%) ^b^
1	NBS	−	CH_2_Cl_2_: CCl_4_	48
2	NBS	−	TFT	20
3	NBS	−	TFE	40
4	NBS	−	DME	40, (36 ^c^) ^d^
5	NBS	−	AcOH	23, (12 ^c^) ^d^
6	NBS	−	MeCN	3, (− ^c^)
7	NBS	−	MeOH	16, (− ^c^)
8	NBS	−	EtOH	19, (6 ^c^)
9	NBS	−	water	4, (− ^c^)
10	NBS	−	DMSO	7
11	NCS	−	DME	33
12	NIS	−	DME	40
**13**	**NBS**	**Bu_4_NBr**	**DME**	**60 ^e^**
14	NBS	Bu_4_NBr	AcOH	46 ^e^
15	NCS	Me_4_NCl	DME	27 ^e^
16	NIS	Bu_4_NI	DME	− ^e,f^
17	NBS	HBr	DME	17 ^e^
18	NBS	LiBr	DME	55 ^e^
19	NBS	Bu_4_NBr	EtOH	28 ^e^

^a^ Reactions carried out on a 1 mmol scale, NXS (1 molar equiv.), in solvent (4 mL), at room temperature, and for 24 h. ^b^ Isolated yield. ^c^ Reaction carried out at reflux. ^d^ Reaction carried out at 0 °C; 25% yield obtained. ^e^ Starting material and source of X^−^ (1 molar equiv.) were stirred together for 5 min prior to addition of NXS (1 molar equiv.). ^f^ Reaction carried out over 3 days. No transformation of starting material.

**Table 2:** Substituent effects on benzothiazole formation via *N*-halosuccinimide-mediated oxidative cyclization.

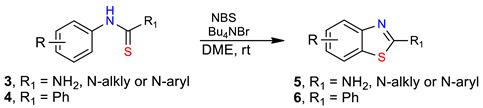
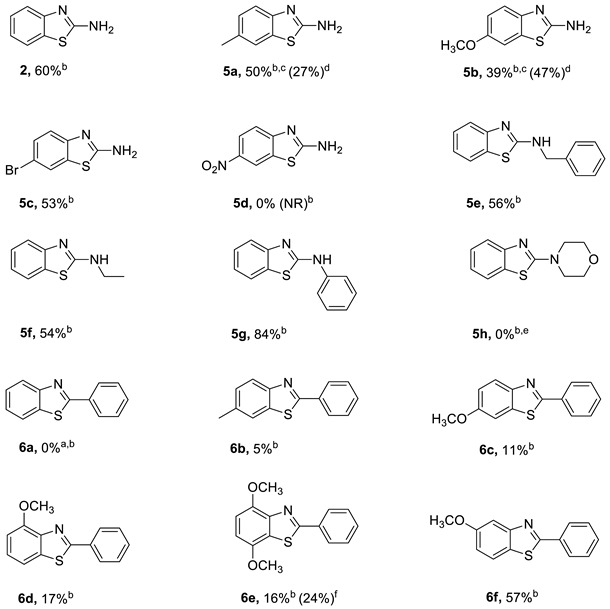

All reactions were carried out on a 1 mmol scale and isolated yields are shown. ^a^ NXS (1 molar equiv.) in solvent (4 mL) at ambient temperature for 24 h. ^b^ Bu_4_NBr (1 molar equiv.) and NBS (1 molar equiv.) in DME (4 mL) at ambient temperature for 24 h. ^c^ Ring-brominated material was also formed as a by-product. ^d^ Me_4_NCl (1 molar equiv.) and NCS (1 molar equiv.) in DME (4 mL) at ambient temperature for 24 h. ^e^ Thioamidoguanidine **7** was formed at 70% yield. No benzothiazole was detected. ^f^ Bu_4_NBr (1 molar equiv.) and NBS (1 molar equiv.) in DME (4 mL) at 60 °C. NR = no reaction.

**Table 3:** One-pot synthesis of 2-aminobenzothiazoles from (iso)thiocyanates ^a^.

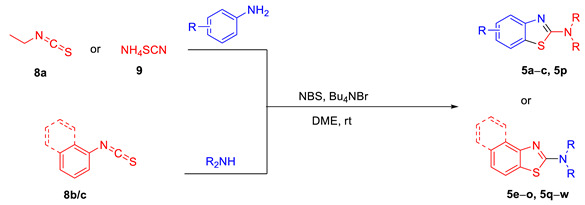
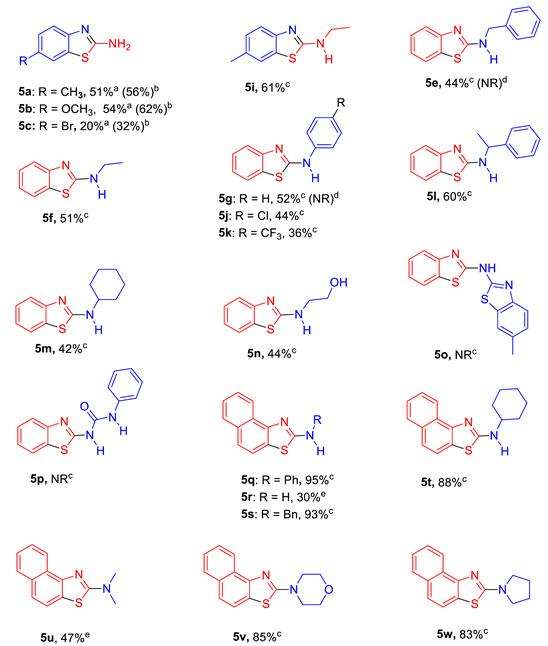

^a^ Isolated yields are shown. Reactions were carried out on a 3 mmol scale, NH_4_SCN (2 molar equiv.), NBS (1 molar equiv.) Bu_4_NBr (1 molar equiv.), in DME (5 mL) at ambient temperature. ^b^ Reactions were carried out on a 3 mmol scale, NH_4_SCN (4 molar equiv.), NBS (1 molar equiv.) Bu_4_NBr (1 molar equiv.), in AcOH (10 mL) at 10 °C to ambient temperature. ^c^ Reaction was carried out on a 1 mmol scale, amine (1 molar equiv.), NBS (1 molar equiv.) Bu_4_NBr (1 molar equiv.), in DME (4 mL) at ambient temperature. ^d^ Reaction was carried out on a 1 mmol scale in AcOH (4 mL) at 10 °C to ambient temperature. ^e^ Reaction carried out on a 1mmol scale with the ammonium salt (1 equiv.), Na_2_CO_3_, (1 equiv.), NBS (1 molar equiv.) Bu_4_NBr (1 molar equiv.), in DME (4 mL) at ambient temperature. NR = no reaction.

**Table 4:** Melting points of benzothiazoles prepared.

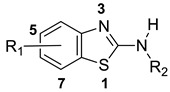				
Compound	R_1_	R_2_	M.P. (°C)	M.P. (°C) [Lit]
**2**	H	H	124–126	128–129 [53]
**5a**	6-CH_3_	H	125–127	129–130 [54]
**5b**	6-OCH_3_	H	163–165	158–156 [54]
**5c**	4-Br	H	199–200	215–217 [28]
**5e**	H	CH_2_Ph	161–162	160–161 [14]
**5f**	H	Et	94–95	114–116 [55]
**5g**	H	Ph	147–149	159–160 [56]
**5i**	6-CH_3_	Et	Viscous oil	104–106 [55]
**5j**	H	4-Cl-Ph	189–191	207–208 [57]
**5k**	H	4-CF_3_-Ph	173–175	194–196 [57]
**5l**	H	CH(CH_3_)Ph	Tan wax	131–132 [14]
**5m**	H	cyclohexyl	Viscous oil	78–80 [57]
**5n**	H	CH_2_CH_2_OH	97–99	99–101 [28]
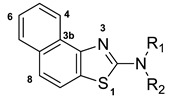				
**Compound**	**R_1_**	**R_2_**	**M.P. (°C)**	**M.P. (°C) [Lit]**
**5q**	H	Ph	139–141	140–141 [42]
**5r**	H	H	188–191	190–192 [18]
**5s**	H	Bn	107–109	104–106 [18]
**5t**	H	cyclohexyl	light brown viscous oil	115–116 [42]
**5u**	CH_3_	CH_3_	116–118	115–117 [43]
**5v**	-(CH_2_)_2_-O-(CH_2_)_2_-		188–190	183–184 [38]
**5w**	-(CH_2_)_4_-		125–127	143–145 [38]
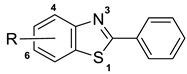				
**Compound**	**R**		**M.P. (°C)**	**M.P. (°C) [Lit]**
**6b**	6-CH_3_		120–122	118–119 [30]
**6c**	6-OCH_3_		113–115	114–116 [30]
**6d**	4-OCH_3_		100–103	99–100 [27]
**6e**	4,7-OCH_3_		119–121	122–124 [30]
**6f**	5-OCH_3_		74–76	75–77 [30]

## Supplementary Materials

The following supporting information can be downloaded at: https://www.mdpi.com/article/10.3390/molecules27227876/s1. Detailed experimental procedures and characterization of compounds.
