# Supplementary material for: A Green Approach to 2-Substituted Benzo- and Naphthothiazoles via N-bromosuccinimide/Bromide-Mediated C(aryl)-S Bond Formation
Source: Molecules. 2022 Nov 15;27(22):7876. doi: 10.3390/molecules27227876 (PMC9697867; doi:10.3390/molecules27227876)

molecules

MDPI

SUPPORTING INFORMATION

A Green Approach to 2-Substituted Benzo- and Naphthothiazoles via N-bromosuccinimide/Bromide-Mediated C(aryl)-S Bond Formation

Ainka T. Brown and Nadale K. Downer-Riley *

The Department of Chemistry, 2 Plymouth Crescent, The University of the West Indies, Mona, Kingston 7, Jamaica
* Correspondence: nadale.downer02@uwimona.edu.jm

Experimental procedures and characterization data.

Table of contents

General information	S2
General procedures for preparation of substrates	S2
Characterization data for substrates	S7
General procedure for benzothiazole synthesis	S7
Procedure for gram scale synthesis	S9
Procedures for control experiments	S9
Characterization data for products	S10
Screening Conditions for Thioamide Cyclization	S17
^{1}H and ^{13}C NMR spectra	S18

Experimental

1. General information

Reagents and solvents were obtained from commercial sources and used as received without further purification. Unless otherwise stated reactions were carried out under an air atmosphere.

AcOH refers to glacial acetic acid; CH_2Cl_2 refers to methylene chloride; DME refers to 1,2-dimethoxyethane; EtOAc refers to ethyl acetate; EtOH refers to ethanol; and NMP refers to *N*-methyl-2-pyrrolidone.

Thin layer chromatography (TLC) was carried out using silica gel pre-coated alumina plates (200 μm) and visualized using ultraviolet light. Column chromatography was performed using silica gel (200-400 mesh).

^{1}H NMR and ^{13}C NMR spectra were recorded in $CDCl_3$, acetone-d_6, or DMSO-d_6 on Bruker Avance 200 and 500 MHz spectrometers. Chemical shifts are reported in parts per million (δ).

Coupling constants (*J*) are reported in hertz (Hz). The following abbreviations for multiplicities are used: s = singlet, br s = broad singlet, d = doublet, t = triplet, q = quartet, m = multiplet.

Melting points were recorded using a Gallenkamp melting point apparatus and open capillary tubes. IR spectra were obtained using a Bruker Vector 22 spectrometer. Known compounds were identified by comparing their characterization data with that in the literature.

2. General procedures for preparation of substrates

2.1. Method for preparation of phenylthioureas 1, 3a-3d (Method A) [44]

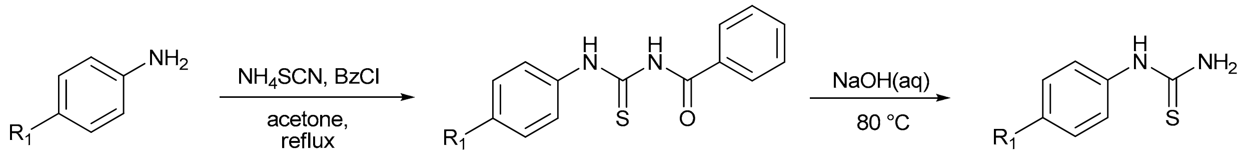

1 R_1 = H
3a R_1 = CH_3
3b R_1 = OCH_3
3c R_1 = Br
3d R_1 = NO_2

Benzoyl chloride (1 molar equiv.) was added in portions with stirring to a 2.2 M solution of ammonium thiocyanate (1.1 molar equiv.) in dry acetone at room temperature followed by heating at reflux for approximately 15 minutes. To this mixture was added in portions a 4.4 M solution of the required aniline (1 molar equiv.) in dry acetone at such a rate to allow for gentle reflux. After stirring for 1 hour the reaction mixture was poured onto ice (~100 g). The precipitated benzoyl thiourea was collected by suction filtration, washed with water and air dried. These solids were then hydrolysed by addition to hot 3 M aqueous sodium hydroxide (1 mL per mmol of benzoylthiourea) and stirring at ~80 °C for 15 minutes. The mixture was filtered hot to remove any insoluble material and the filtrate cooled to room temperature. The pH was adjusted by acidifying with concentrated hydrochloric acid and then making slightly basic (~ pH 8) with concentrated aqueous ammonia. The precipitated phenylthiourea was collected by suction filtration and purified silica gel column chromatography using hexane – EtOAc as eluent.

2.2. Synthesis of N-substituted phenylthioureas 3e – 3h (Method B)

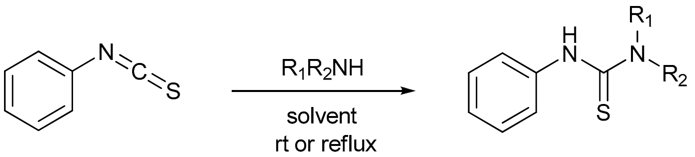

3e R_1 = H, R_2 = Bn
3f R_1 = H, R_2 = Et
3g R_1 = H, R_2 = Ph
3h R_1 = R_2 = -$(CH_2)_2O(CH_2)_2$-

Thioureas **3e** – **h**, were prepared using known procedures. [45,46]

The required amine (1.3 molar equiv.) was added slowly to a solution of phenylisothiocyanate (1 molar equiv.) in 15 mL of solvent (EtOAc, EtOH or CH_2Cl_2) and the resulting solution stirred at room temperature or heated at reflux until tlc indicated the consumption of starting material. If the product precipitated it was collected by suction filtration, otherwise the solvent was removed under reduced pressure and the residue triturated with cold EtOH or hexane to give solids which were collected by suction filtration and used without further purification.

2.3. Preparation of thiobenzanilides 4a – 4c (Method C)

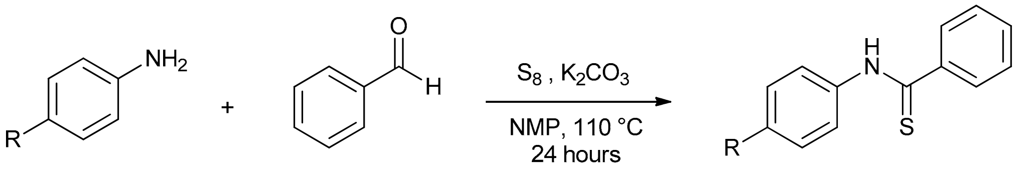

4a R = H

4b R = CH_3

4c R = OCH_3

Thiobenzanilides **4a – 4c** were prepared using modified conditions of a reported procedure. [47,48]

Potassium carbonate (2 molar equiv.) and sulfur (3 molar equiv.) were thoroughly combined in a Smith process vial. NMP (3 mL/mmol of aniline) was introduced followed by the aniline (1 molar equiv.) and the suspension vigorously stirred at room temperature until it became blue. Benzaldehyde (1.5 molar equiv.) was then added and the vial sealed with a Teflon cap and aluminium crimp. The reaction mixture was heated with vigorous stirring at 110 °C for 24 hours. On cooling to room temperature, the mixture was carefully poured onto crushed ice (100 – 200 g) and left to stand until the product precipitated. Collection by suction filtration and purification by silica gel column chromatography using hexane – EtOAc as eluent gave the products as yellow solids.

2.4. Preparation of thiobenzanilides 4d-4f (Method D) [27]

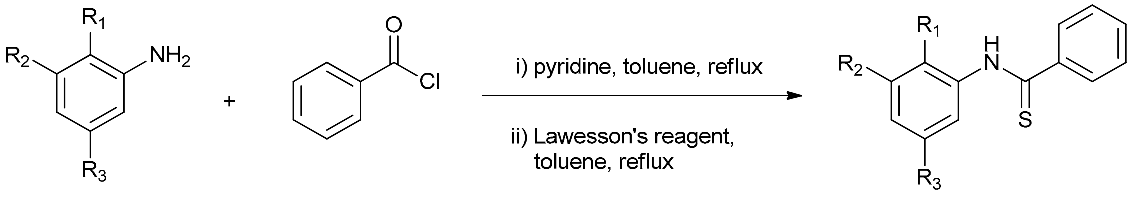

4d R_1= OCH_3, R_2 = H, R_3 = H

4e R_1= OCH_3, R_2 = H, R_3 = OCH_3

4f R_1 = H, R_2 = OCH_3, R_3 = H

Benzoyl chloride (1.2 molar equiv.) was added to a solution of the aniline (1 molar equiv.) in dry toluene (1.5 mL/ mmol of aniline) and pyridine (0.7 mL/mmol of aniline) followed by heating at reflux under a nitrogen atmosphere for 2 hours. The mixture was cooled to room temperature and then poured into cold 1M hydrochloric acid (50 – 100 mL) and extracted with EtOAc (3 × 15 mL). The combined organic solutions were washed with 1 M hydrochloric acid and saturated aqueous sodium bicarbonate, dried over magnesium sulfate and concentrated under reduced pressure. Purification of the crude material (silica gel chromatography, hexane – EtOAc) gave the required amide. Lawesson's reagent (0.6 molar equiv.) was then added to a 0.1 M solution of the amide in dry toluene followed by heating at reflux under a nitrogen atmosphere for 4 - 6 hours. Concentration under reduced pressure and purification by silica gel column chromatography using hexane-CH_2Cl_2 (1:1) as eluent gave the desired thiobenzanilides.

3. Characterization data for substrates

Phenylthiourea (1)

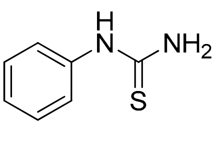

Yield: 4.93 g, 70% from aniline (Method A), The product was purified by chromatography, hexane – EtOAc, 1:1; off-white solids; Mp 140.0 – 142.3°C, (Lit. 153-154 °C)[44]; IR (ATR) ν/cm^{-1} 3423, 3167, 1654, 1608, 1517, 1445, 1313, 1260, 1230, 809; ^{1}H NMR (200 MHz, DMSO-d$_6$) δ/ppm 9.65 (1H, br s, NH), 7.35-7.14 (7H, m, Ar-H, NH$_2$); ^{13}C NMR (50 MHz, DMSO-d$_6$) δ/ppm 181.1, 138.3, 128.9, 125.3, 123.7.

4-methylphenylthiourea (3a)

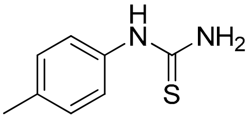

Yield 1.37 g, 83 % from *p*-toluidine (Method A), The product was purified by chromatography, hexane – EtOAc, 2:1; cream solids; Mp 173.5 – 174.9 °C, (Lit. 185 – 186 °C)[50]; IR (ATR) ν/cm^{-1} 3306, 3163, 1645, 1612, 1531, 1493, 1462, 1311, 1288, 1264, 1235, 1070, 802; ^{1}H NMR (500 MHz, DMSO-d$_6$) δ/ppm 9.57 (1H. br s , NH), ^{13}C NMR (125 MHz, DMSO-d$_6$) δ/ppm 180.8, 136.0, 134.7, 130.0, 124.7, 20.8.

4-methoxyphenylthiourea (3b)

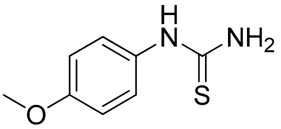

Yield 1.56 g, 86% from *p*-anisidine (Method A), The product was purified by chromatography, hexane – EtOAc, 4:1; grey solids; Mp 208.0-209.6 °C. (Lit. 206-209 °C)[51]; IR (ATR) ν/cm^{-1} 3401, 3134, 3013, 1646, 1622, 1531, 1513, 1466, 1242, 1077, 1018, 827; ^{1}H NMR (500 MHz, DMSO-d$_6$) δ/ppm 7.13 (2H, m, 2-H, 6-H), 6.90 (2H, d, J = 8.5 Hz, 3-H, 5-H), 3.70 (3H, s, OCH$_3$), ^{13}C NMR (125 MHz, DMSO-d$_6$) δ/ppm 181.5, 156.5, 129.1, 127.7, 123.4, 114.3, 55.8.

4-bromophenylthiourea (3c)

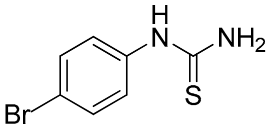

Yield: 1.44 g, 67% from *p*-bromoaniline (Method A), The product was purified by chromatography, hexane – EtOAc, 2:1; cream solids; Mp 172.8 – 175.4 °C (Lit. 142-144 °C)[52]; IR (ATR) ν/cm^{-1} 3227, 3125, 1618, 1510, 1489, 1396, 1297, 1230, 1058, 1010, 801; ^{1}H NMR (500 MHz, acetone-d$_6$) δ/ppm 9.33 (1H, br s, NH); 7.51 (4H, m, Ar-H), 7.13 (2H, br s, NH$_2$); ^{13}C NMR (125 MHz, acetone-d$_6$) δ/ppm 182.6, 138.6, 131.7, 122.1, 117.3.

4-nitrophenylthiourea (3d)

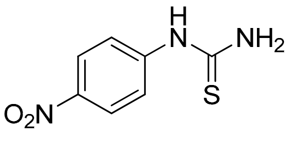

Yield: 0.15 g, 42% from *p*-nitroaniline (Method A), The product was purified by chromatography, hexane – EtOAc, 4:1; light yellow solids; Mp 206.0 – 208.1 °C, (Lit. 213 - 215 °C)[51]; IR (ATR) ν/cm^{-1} 3481, 3357, 3217, 1629, 1586, 1469, 1298, 1181, 1111, 839; ^{1}H NMR (500 MHz, DMSO-d$_6$) 10.21 (1H, s, NH); 8.04 (2H, d, J = 8.0 Hz, 3-H, 5-H), 7.68 (2H, d, J = 8.5 Hz, 2-H, 6-H), ^{13}C NMR (125 MHz, DMSO-d$_6$) δ/ppm 181.5, 145.6, 143.2, 124.7, 122.2.

1-benzyl-3-phenylthiourea (3e)

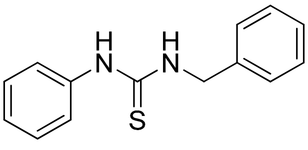

Yield: 1.87 g, 84% (Method B), white solids; Mp 151.3 -151.6 °C, (Lit. 153-154 °C)[14]; IR (ATR) ν/cm^{-1} 3364, 3144, 2975, 1537, 1507, 1298, 1245, 1177, 872; ^{1}H NMR (500 MHz, $CDCl_3$) δ/ppm 8.54 (1H, br s, NH), 7.44-7.39 (2H, m, Ar-H), 7.36-7.28, 6H, m, Ar-H), 7.24 (2H, d, J = 7.5 Hz, Ar-H), 6.37 (1H, br s, NH), 4.89-4.88 (2H, m, CH_2); ^{13}C NMR (125 MHz, $CDCl_3$) δ/ppm 180.8, 137.3, 136.1, 130.2, 128.8, 127.8, 127.7, 127.3, 125.3, 49.4.

1-ethyl-3-phenylthiourea (3f)

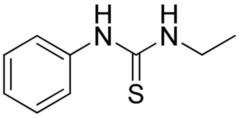

Yield: 1.29 g, 97% (Method B), white solids; Mp 100.0 -101.4 °C, (Lit. 100 -101 °C)[45]; IR (ATR) ν/cm^{-1} 3233, 1537, 1519, 1493, 1318, 1241, 1203, 1052, 798, 718; ^{1}H NMR (200 MHz, $CDCl_3$) δ/ppm 8.54 (1H, br s, NH), 7.41-7.34 (2H, m, H-2,6), 7.27-7.17 (3H, m, H-3,4,5), 6.10 (1H, br s, NH), 3.61 (2H, m), 1.14 (3H, t, J = 7.2 Hz, CH_3); ^{13}C NMR (125 MHz, $CDCl_3$) δ/ppm 180.0, 136.5, 130.0, 129.5, 126.9, 125.1, 123.6, 40.2, 14.3

1,3-diphenylthiourea (3g)

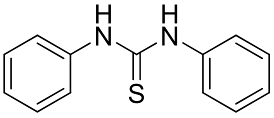

Yield: 4.19 g, 88% (Method B), white solids; Mp 151.3 -151.5 °C, (Lit. 157 °C)[46]; IR (ATR) ν/cm^{-1} 1588, 1548, 1521, 1492, 1345, 1292, 1272, 1173, 1022, 934; ^{1}H NMR (500 MHz, $CDCl_3$) δ/ppm 8.21 (2H, br s, NH), 7.42-7.41 (10H, m, Ar-H); ^{13}C NMR (125 MHz, $CDCl_3$) δ/ppm 179.8, 137.2, 129.6, 127.1, 125.3.

***N*-phenylmorpholine-4-carbothioamide (3h)**

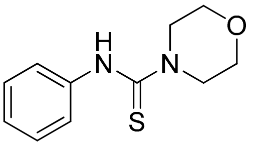

Yield: 0.47 g, 71% (Method B), white solids; Mp 132.0 – 133.7°C, (Lit. 136 -137 °C)[14]; IR (ATR) ν/cm^{-1} 3149, 1593, 1530, 1412, 1322, 1306, 1220, 1208, 1111, 1028, 941, 855; ^{1}H NMR (200 MHz, $CDCl_3$) δ/ppm 7.46 (1H, br s, NH), 7.37 – 7.27 (2H, m, 2-H, 6-H), 7.19-7.10 (3H, m, 3-H, 4-H, 5-H), 3.82-3.77 (4H, m, -C*H*2-O-), 3.73-.3.68 (4H, m, -C*H*2-N-); ^{13}C NMR (50 MHz, $CDCl_3$) δ/ppm 184.1, 140.0, 129.1, 125.3, 123.0, 66.0, 49.7.

***N*-phenylbenzothioamide (4a)**

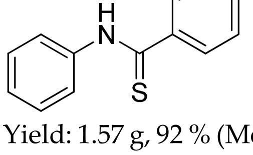

Yield: 1.57 g, 92 % (Method C), The product was purified by chromatography, hexane – EtOAc, 5:1; yellow solids; Mp 97.6 – 99.2°C, (Lit. 97-98 °C)[48]; IR (ATR) ν/cm^{-1} 3012, 1595, 1546, 1495, 1445, 1367, 1240, 1218, 991, 758, 714; ^{1}H NMR (500 MHz, $CDCl_3$) δ/ppm 9.13 (1H, br s, NH); 7.86-7.77 (5H, m, 2-6-H), 7.52 -7.29 (5H, m, 2'-6'-H); ^{13}C NMR (125 MHz, $CDCl_3$) δ/ppm 198.5, 143.0, 139.0, 131.3, 129.1, 128.6, 127.0, 126.8, 123.9.

***N*-(4-methylphenyl)benzothioamide (4b)**

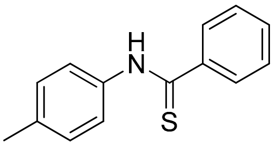

Yield: 0.71 g, 52% (Method C), The product was purified by chromatography, hexane – EtOAc, 6:1; yellow solids; Mp 128.2 -129.0 °C, (Lit. 129-130 °C)[48]; IR (ATR) ν/cm^{-1} 3171, 2990, 1518, 1448, 1352, 1207, 989; ^{1}H NMR (500 MHz, $CDCl_3$) δ/ppm 9.03 (1H, br s , NH), 7.88 (2H, d, J = 7.5 Hz, 2-H, 6-H), 7.64 (2H, d, J = 7.5 Hz, 2'-H, 6'-H), 7.53-7.45 (3H, m, 3-5-H), 7.27 (2H, m, 3'-H,5'-H), 2.41 (3H, s, CH_3); ^{13}C NMR (125 MHz, $CDCl_3$) δ/ppm 198.4, 143.1, 137.1, 136.5, 131.3, 129.6, 128.6, 126.8, 123.9, 21.3.

***N*-(4-methoxyphenyl)benzothioamide (4c)**

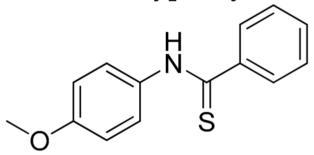

Yield: 0.67 g, 56% (Method C). The product was purified by chromatography, hexane – EtOAc, 8:1, yellow solids; Mp 130.1 -132.0 °C, (Lit. 129-130 °C)[30]; IR (ATR) ν/cm^{-1} 3164, 2991, 1598, 1513, 1463, 1446, 1357, 1302, 1247, 1176, 1040, 988, 824; ^{1}H NMR (500 MHz, $CDCl_3$) δ/ppm 8.99 (1H, br s, NH), 7.89 (2H, d, J = 7.5 Hz, 2',6'-H), 7.67 (2H, d, J = 8 Hz, 2,6-H), 7.55-7.45 (3H, m, 3-5-H), 6.99 (2H, d, J = 6 Hz, 3'-H, 5'- H), 3.87 (3H, s, OCH_3); ^{13}C NMR (125 MHz, $CDCl_3$) δ/ppm 198.7, 158.3, 142.8, 132.1, 131.2, 128.8, 126.9, 125.7, 114.2, 55.7.

***N*-(2-methoxyphenyl)benzothioamide (4d)**

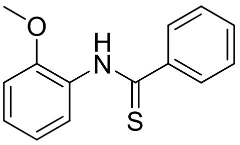

Yield: 0.68 g, 93% from *o*-anisidine (Method D), yellow solids; ; Mp 76.7 – 78.9 °C, (Lit. 78-79)[30]; IR (ATR) ν/cm^{-1} 3363, 1598, 1520, 1483, 1446, 1369, 1290, 1247, 1178, 1044, 1021, 990, 837; ^{1}H NMR (500 MHz, $CDCl_3$) δ/ppm 9.71 (1H, br s, NH), 9.18 (1H, s, 6'-H), 7.90 (2H, m, 2,6-H), 7.52-7.47 (3H, m, 3-5-H), 7.26 (1H, s, 4'-H), 7.09-7.00 (2H, m, 3',5'-H), 3.93 (3H, s, OCH_3); ^{13}C NMR (125 MHz, $CDCl_3$) δ/ppm 196.1, 150.0, 144.0, 131.1, 128.9, 128.7, 126.8, 126.7, 121.6, 120.4, 110.5, 56.1.

***N*-(2,5-dimethoxyphenyl)benzothioamide (4e)**

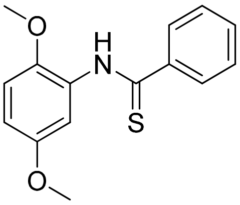

Yield 1.15 g, 90% from 2,5-dimethoxyaniline (Method D), yellow solids; Mp 61.5 - 63.0 °C, (Lit. 61 – 62 °C)[30]; IR (ATR) ν/cm^{-1} 3364, 1599, 1533, 1447, 1367, 1230, 1127, 1043, 1018, 998, 795, 691; ^{1}H NMR (500 MHz, $CDCl_3$) δ/ppm 9.78 (1H, br s , NH), 9.12 (1H, s, 6'-H), 7.88 (2H, m, 2-H, 6-H), 7.52-7.47 (3H, m, 3-5-H), 6.91 (1H, d, J = 8.5 Hz, 4'-H), 6.78 (1H, d, J = 7.5 Hz, 3'-H), 3.91 (3H, s, OCH_3), 3.85 (3H, s, OCH_3), ^{13}C NMR (125 MHz, $CDCl_3$) δ/ppm 195.8, 153.1, 144.2, 143.8, 131.0, 129.3, 128.7, 126.7, 111.4, 111.0, 107.0, 56.4, 55.9.

***N*-(3-methoxyphenyl)benzothioamide (4f)**

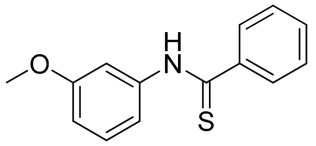

Yield:1.00 g, 87% from *m*-anisidine (Method D), yellow solids; Mp 76.0-78.0 °C, (Lit. 81 -82 °C);[30] IR (ATR) ν/cm^{-1} 3363, 2929, 1650, 1600, 1520, 1491, 1463, 1367, 1292, 1249, 1156, 1044, 1023, 991, 845; ^{1}H NMR (500 MHz, $CDCl_3$) δ/ppm 9.19 (1H, br s, NH), 7.82-7.81 (2H, m, 2,6-H), 7.58 (1H, m, 6'-H), 7.50-7.49 (1H, m, 4-H), 7.42 (2H, m, 3-H,5-H), 7.32 (1H, m, 5'-H), 7.23 (1H, m, 4'-H), 6.84 (1H, m, 2'-H), 3.82 (3H, s, OCH_3); ^{13}C NMR (125 MHz, $CDCl_3$) δ/ppm 198.3, 160.0, 143.2, 140.2, 131.3, 129.8, 128.6, 126.8, 115.8, 112.8, 109.2, 55.5.

4. General procedures for preparation of benzothiazoles

*4.1. Intramolecular cyclisation of phenylthioureas and thiobenzanilides mediated by N-halosuccinimide (NXS) and quaternary ammonium halides (R_4NX) (**Method E**)*

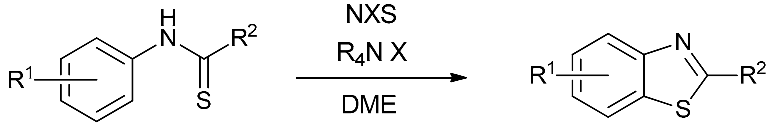

2, 5a-c, 5e-g, 6b-f

The phenylthiourea or thiobenzanilide substrate was either dissolved to give a 0.25 M solution or suspended in DME. Quaternary ammonium halide (1 molar equiv.) was then added. Care was taken to exclude moisture. After stirring for approximately 5 minutes, *N*-halosuccinmide (1 molar equiv.) was added in portions. The reaction was vigorously stirred at ambient temperature until TLC indicated the consumption of starting material (4 -24 hours) following which it was slowly poured onto crushed ice (50 g). Concentrated aqueous ammonia was then used to adjust the pH to ~10. The precipitated solids were collected and dried by suction filtration. If precipitation did not occur then the aqueous phase was extracted with EtOAc (3 x 15 mL), the organic phase washed with brine (15 mL), dried over magnesium sulfate, and concentrated under reduced pressure. Purification of the crude material was done using column chromatography with hexane – EtOAc or hexane - CH_2Cl_2 as eluent.

*4.2. One-pot synthesis of 2-aminobenzothiazoles using ammonium thiocyanate, N-bromosuccinimide (NBS) and tetrabutylammonium bromide (Bu_4NBr) (**Method F**)*

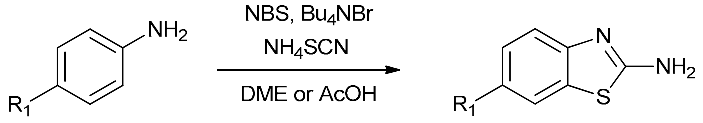

5a $R_1 = CH_3$
5b $R_1 = OCH_3$
5c $R_1 = Br$

4.2.1. Using DME as solvent:

A solution of the aniline (3 mmol), ammonium thiocyanate (2 molar equiv.) and Bu_4NBr (1 molar equiv.) in DME (5 mL) was stirred at room temperature for 5 minutes. Care was taken to exclude moisture. *N*-bromosuccinimide (1 molar equiv.) was then added in portions. After stirring vigorously at ambient temperature for until TLC indicated the consumption of starting material (5 -24 hours) the mixture was poured onto crushed ice (50 g) and made basic (pH ~10) using concentrated aqueous ammonia. The precipitated solids were collected and dried by suction filtration. If precipitation did not

occur readily then extraction with EtOAc (3 x 20 mL) was carried out. The organic phase was washed with brine (20 mL), dried over magnesium sulfate, and concentrated under reduced pressure. Purification of the crude material was done using column chromatography with hexane – EtOAc as eluent.

4.2.2. Using glacial acetic acid as solvent:

A solution of aniline (3 mmol) and ammonium thiocyanate (12 mmol) in glacial AcOH (10 mL) was stirred at ambient temperature for 10 minutes. Care was taken to exclude moisture. The mixture was cooled to 10 °C and Bu_4NBr (3 mmol) introduced to give a suspension. *N*-bromosuccinimide (3 mmol) was then added in portions over 10 minutes whilst still cooling. The reaction was allowed to gradually warm to ambient temperature at which it was stirred for 24 hours. The mixture was poured into an ice and water mixture (~150 mL) and the pH adjusted to ~10 using concentrated aqueous ammonia. The precipitated solids were collected and dried by suction filtration. If precipitation did not occur readily then extraction with EtOAc (3 x 20 mL) was carried out. The organic phase was washed with brine (20 mL), dried over magnesium sulfate, and concentrated under reduced pressure. Purification of the crude material was done using column chromatography with hexane – EtOAc as eluent.

4.3. One-pot synthesis of 2-aminobenzothiazoles using isothiocyanate, N-bromosuccinimide (NBS) and tetrabutylammonium bromide (Bu_4NBr) (Method G)

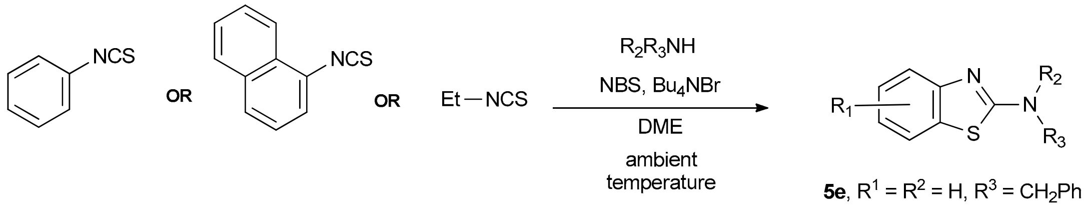

5e, $R^1 = R^2 = H$, $R^3 = CH_2Ph$
5f, $R^1 = R^2 = H$, $R^3 = Et$
5g, $R^1 = R^2 = H$, $R^3 = Ph$
5i, $R^1 = 4\text{-}CH_3$, $R^2 = H$, $R^3 = Et$
5j, $R^1 = R^2 = H$, $R^3 = 4\text{-Cl-Ph}$
5k, $R^1 = R^2 = H$, $R^3 = 4\text{-}CF_3\text{-Ph}$
5l, $R^1 = R^2 = H$, $R^3 = CH(CH_3)Ph$
5m, $R^1 = R^2 = H$, R^3 = cyclohexyl
5n, $R^1 = R^2 = H$, $R^3 = CH_2CH_2OH$
5q, $R^1 = Ph$, $R^2 = H$, $R^3 = Ph$
5r, $R^1 = Ph$, $R^2 = H$, $R^3 = H$
5s, $R^1 = Ph$, $R^2 = H$, $R^3 = Bn$
5t, $R^1 = Ph$, $R^2 = H$, R^3 = cyclohexyl
5u, $R^1 = Ph$, $R^2 = R^3 = CH_3$
5v, $R^1 = Ph$, $R^2 = R^3 = -(CH_2)_2\text{-O-}(CH_2)_2-$
5w, $R^1 = Ph$, $R^2 = R^3 = -(CH_2)_4-$

A solution of isothiocyanate (1 mmol) and Bu_4NBr (1 mmol) in DME (4 mL) was stirred at ambient temperature for 5 minutes. Care was taken to exclude moisture. Amine (1.1 mmol) was then added slowly and the reaction mixture allowed to stir until isothiocyanate was consumed as determined by TLC (45 minutes to 2 hours). *N*-bromosuccinimide (1 mmol) was then added in portions and the mixture allowed to stir vigorously at ambient temperature. After TLC indicated the consumption of the intermediate compound and the formation of product (1-20) hours the mixture was poured onto crushed ice (50 g) and made basic (pH ~10) using concentrated aqueous ammonia. The precipitated solids were collected and dried by suction filtration. If precipitation did not occur readily then the aqueous phase was extracted with EtOAc (3 x 15 mL). The organic phase was washed with brine (15 mL), dried over magnesium sulfate, and concentrated under

reduced pressure. Purification of the crude material was done using either recrystallization ($EtOH-H_2O$) or column chromatography with hexane – EtOAc as eluent.

5. Procedure for gram scale synthesis

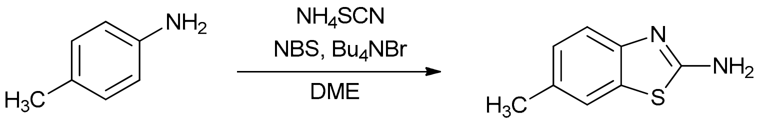

7a **5a**

A solution of *p*-toluidine (15 mmol), ammonium thiocyanate (2 molar equiv.) and Bu_4NBr (1 molar equiv.) in DME (15 mL) was stirred at room temperature for 5 minutes. Care was taken to exclude moisture. *N*-bromosuccinimide (1 molar equiv.) was then added in portions. After stirring vigorously at ambient temperature for 24 hours the mixture was poured onto crushed ice (120 g) and made basic (pH ~10) using concentrated aqueous ammonia. Extraction with EtOAc (3 x 30 mL) was carried out. The organic phase was washed with brine (30 mL), dried over magnesium sulfate, and concentrated under reduced pressure. Purification of the crude material was done using column chromatography with hexane – EtOAc (2:1) as eluent to give benzothiazole **5a** (1.79 g, 72%).

6. Procedures for control experiments

I)

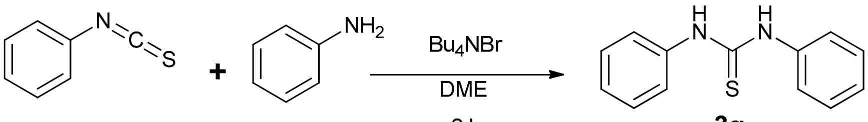

8b 2 h **3g**

Aniline (0.1 mL, 1.1 mmol) was added dropwise to a mixture Bu_4NBr (1 mmol) and phenylisothiocyanate (**8b**) (0.12 mL, 1 mmol) in DME (4 mL). Care was taken to exclude moisture. The resulting solution was stirred vigorously at ambient temperature for 2 h at which point TLC indicated the consumption of starting material. The solvent was removed under reduced pressure and the residue triturated with cold MeOH-hexane. The precipitated white, crystalline solids were collected and dried by suction filtration to give 0.21 g (90%) of 1,3-diphenylthiourea **3g**.

II)

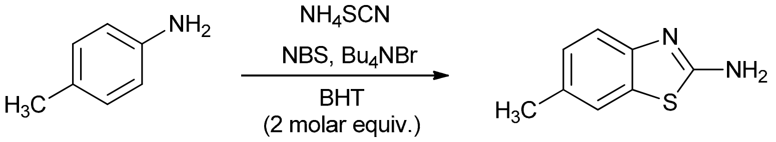

6a DME **5a**

BHT (1.33 g, 6 mmol), *p*-toluidine (0.33 g, 3 mmol), ammonium thiocyanate (0.47 g, 6 mmol) and Bu_4NBr (0.968 g, 3 mmol) were combined in DME (5 mL). Care was taken to exclude moisture. This mixture was stirred for approximately 1 minute following which *N*-bromosuccinimide (0.54 g, 3 mmol) was added in portions. The reaction was vigorously stirred at ambient temperature for 24 hours then poured into cold water (50 mL). The aqueous phase was made basic (~pH 10) by the addition of concentrated ammonia. Extraction with EtOAc (3 x 15 mL), drying of the combined organic solutions over sodium sulfate, and concentrating under reduced pressure gave crude material. Purification was carried out using silica-gel chromatography (hexane-EtOAc 4:1 → 2:1) to give benzothiazole **5a** (0.32 g, 64%).

III)

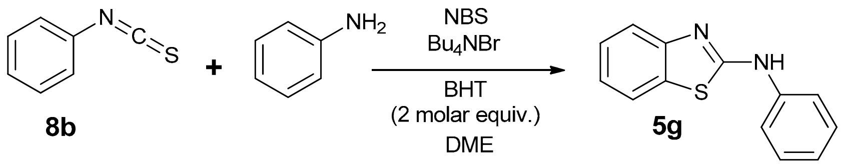

Aniline (0.1 mL, 1.1 mmol) was added dropwise to a vigorously stirred mixture of phenylisothiocyanate (**8b**) (0.12 mL, 1 mmol), Bu_4NBr (0.33 g, 1 mmol) and BHT (0.45 g, 2 mmol) in DME (4 mL). Care was taken to exclude moisture. The resulting solution was stirred until TLC indicated the consumption of starting material and formation of 1,3-diphenylthiourea (**3g**). *N*-bromosuccinimide (0.18 g, 1 mmol) was then added in portions and the reaction mixture stirred vigorously at ambient temperature. After 24 hours the solution was poured into cold water (50 mL) and the aqueous phase made basic (~pH 10) with concentrated ammonia. Crude material was obtained by extraction with EtOAc (3 x 15 mL), washing of the combined organic solutions with brine, drying over sodium sulfate and concentrating under reduced pressure. Chromatographic purification using hexane – EtOAc, 5:1 → 4:1 as eluent gave 0.12 g (54%) of benzothiazole **5g**.

7. Characterisation data for benzothiazoles and naphthothiazoles

2-aminobenzothiazole (2)

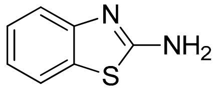

The product was purified by chromatography, hexane – EtOAc, 3:2. Yield: 0.09 g, 60 % (Method E, using NBS/ Bu_4NBr), cream solids; Mp 124.0 – 126.7 °C (Lit. 128 – 129 °C)[53]; IR (ATR) ν/cm^{-1} 1569, 1530, 1444, 1363; ^{1}H NMR (500 MHz, $CDCl_3$) δ/ppm 7.33 – 7.29 (2H, m, Ar-H); 7.27 -7.01 (2H, m, Ar-H), 5.88 (br s, NH_2), ^{13}C NMR (125 MHz, $CDCl_3$) δ/ppm 168.6, 146.7, 137.8, 131.0, 129.1, 123.6, 119.1.

2-amino-6-methylbenzothiazole (5a)

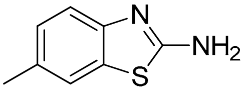

The product was purified by chromatography, hexane – EtOAc, 2:1.

Yield: 0.08g, 50% (Method E, using NBS/ Bu_4NBr);

0.04 g, 27% (Method E, using NCS/ $Me_4N^+Cl^-$);

0.24 g, 51% (Method F, using DME as solvent, 3 mmol scale);

1.79 g, 72% (Method F, using DME as solvent, 15 mmol scale);

0.27 g, 56% (Method F, using AcOH as solvent, 3 mmol scale).

Cream solids; Mp 125.0 – 127.2 °C (Lit. 129 – 130 °C)[54]; IR ν/cm^{-1} (ATR) 3392, 1620, 1537, 1462, 1279, 808; ^{1}H NMR (200 MHz, $CDCl_3$) δ/ppm 7.33-7.27 (2H, m, 4-H,7-H), 7.00 (1H, d, *J* = 7.4 Hz 5-H), 5.27 (2H, br s, NH_2), 2.29 (3H, s, CH_3); ^{13}C NMR (50 MHz, $CDCl_3$) δ/ppm 165.6, 149.8, 132.0, 131.6, 127.1, 120.9, 118.9, 21.1.

2-amino-6-methoxybenzothiazole (5b)

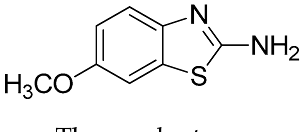

The product was purified by chromatography, hexane – EtOAc 1:1 →2:3.

Yield: 0.07 g, 39% (Method E, using NBS/ Bu_4NBr);

0.08 g, 47 % (Method E, using NCS/ Me_4NCl);

0.30 g, 54% (Method F, using DME as solvent, 3 mmol scale);

0.33 g, 62% (Method F, using AcOH as solvent, 3 mmol scale).

Grey solids; Mp 163.2 – 165.4 °C (Lit. 158 – 159 °C)[54]; IR ν/cm^{-1} (ATR) 3382, 1640, 1546, 1462, 1276, 1205, 1053, 1021, 806; ^{1}H NMR (500 MHz, DMSO-d$_6$) δ/ppm 7.25 (1H, d, *J*= 10 Hz, 4-H), 7.21 (1H, s, 7-H), 7.11 (2H, br s, NH_2), 6.82 (1H, d, *J* = 9 Hz, 5-H), 3.68 (3H, s, OCH_3); ^{13}C NMR (125 MHz, DMSO-d$_6$) δ/ppm 165.8, 154.4, 145.5, 131.3, 118.1, 113.2, 105.4, 55.5.

2-amino-6-bromobenzothiazole (5c)

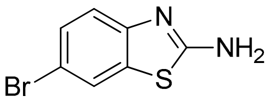

The product was purified by chromatography, hexane – EtOAc, 3:1.

Yield: 0.12 g, 53% (Method E, using NBS/ Bu_4NBr);

0.15 g, 20 % (Method F, using DME as solvent, 3 mmol scale);

0.21 g, 32% (Method F, using AcOH as solvent, 3 mmol scale).

Cream solids; Mp 198.7 - 200 °C, (Lit 215-217 °C)[28]; IR ν/cm^{-1} (ATR) 3456, 1629, 1524, 1442, 1304, 1115, 810; ^{1}H NMR (500 MHz, acetone-d$_6$) δ/ppm 7.84 (1H, s, 7-H), 7.39 (1H, d, *J* = 8 Hz, 5-H), 7.33 (1H, d, *J* = 8 Hz, 4-H), 7.11 (2H, br s, NH_2); ^{13}C NMR (125 MHz, acetone-d$_6$) δ/ppm 167.3, 152.2,133.8, 128.5, 123.2, 119.7, 112.9.

***N*-benzyl-1,3-benzothiazol-2-amine (5e)**

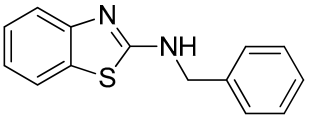

The product was purified by chromatography, hexane – EtOAc 5:1.

Yield: 0.13 g, 56%, (Method E, using NBS/ Bu_4NBr),

0.10 g, 44 % (Method G).

White solids; Mp 160.7 -161.9 °C (Lit 160-161 °C)[14]; IR ν/cm^{-1} (ATR) 2920, 1615, 1572, 1447, 1354, 1236, 1091, 885; ^{1}H NMR (500 MHz, $CDCl_3$) δ/ppm 7.61-7.59 (1H, m, Ar-H), 7.47-7.29 (7H, m, Ar-H), 7.13-7.10 (1H, m, Ar-H), 6.57 (1H, br s , NH), 4.67 (2H, s, CH_2); ^{13}C NMR (125 MHz, $CDCl_3$) δ/ppm 167.8, 152.3, 137.5, 129.1, 128.9, 127.9, 127.7, 126.0, 121.6, 120.9, 118.9, 49.5.

***N*-ethyl-1,3-benzothiazol-2-amine (5f)**

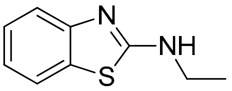

The product was purified by chromatography, hexane – EtOAc 2:1.

Yield: 0.09 g, 54% (Method E, using NBS/ Bu_4NBr)

0.08 g, 51% (Method G)

White solids; Mp 93.6 -95.2 °C (Lit. 114-116 °C)[55]; IR ν/cm^{-1} (ATR) 1608, 1567, 1444, 1266; ^{1}H NMR (200 MHz, $CDCl_3$) δ/ppm 7.62-7.53 (2H, m, 4-H,7-H), 7.35-7.32 (2H, m, 5-H, NH), 7.08 (1 H, t, *J* = 7.4 Hz, 6-H), 3.44 (2H, q, *J*= 7.2 Hz, CH_2), 1.32 (3H, t, *J*= 7.2 Hz, CH_3); ^{13}C NMR (50 MHz, $CDCl_3$) δ/ppm 168.2, 152.5, 130.3, 125.9, 121.2, 120.8, 118.5, 40.4, 14.9.

***N*-phenyl-1,3-benzothiazol-2-amine (5g)**

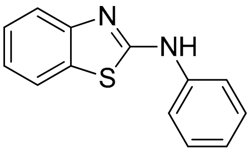

The product was purified by chromatography, hexane – EtOAc 6:1.

Yield: 0.19 g, 84 % (Method E, using NBS/ Bu_4NBr);

0.12 g, 52% (Method G).

Cream solids; Mp 147.1 – 149.0 °C (Lit. 159-160 °C)[56]; IR ν/cm^{-1} (ATR) 1623, 1569, 1446, 1248; ^{1}H NMR (500 MHz, acetone-d$_6$) δ/ppm 7.89 (2H, d, J = 8 Hz, 5-H, 7-H), 7.75 (1H, d, J = 7.5 Hz, 6-H), 7.66 (1H, d, J = 8 Hz, 4-H), 7.34 – 7.40 (3H, m, 2', 4', 6'-H), 7.18 (1H, t, J = 7.5 Hz, 3'-H), 7.07 (1H, t, J = 7.5 Hz, 5'-H), ^{13}C NMR (125 MHz, acetone-d$_6$) δ/ppm 162.0, 152.7, 140.9, 129.1, 128.9, 125.9, 122.5, 122.3, 120.8, 119.9, 118.2.

***N*-ethyl-6-methyl-1,3-benzothiazol-2-amine (5i)**

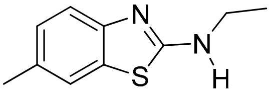

The product was purified by chromatography, hexane – EtOAc 5:1.

Yield: 0.08 g, 41%, viscous tan oil; (Lit. mp. 104-106 °C)[55]; IR ν/cm^{-1} (ATR) 3211, 2970, 1606, 1674, 1647, 1464, 1202, 812; ^{1}H NMR (500 MHz, $CDCl_3$) δ/ppm 7.41-7.43 (2H, m, 5-H, 7-H), 7.11 (1H, d, J = 8.1 Hz, 4-H), 5.64 (1 H, br s, NH), 3.46 (2H, q, *J*= 6.3 Hz, CH_2), 2.39 (3H, s, Ar-CH_3), 1.33 (3H, t, CH_3); ^{13}C NMR (125 MHz, $CDCl_3$) δ/ppm 167.0, 150.3, 131.1, 130.4, 127.1, 120.9, 118.3, 40.3, 21.2, 14.9.

***N*-(4-chlorophenyl)-1,3-benzothiazol-2-amine (5j)**

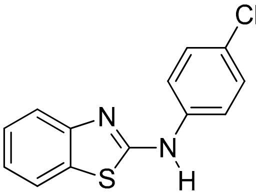

The product was purified by recrystallization from methanol-water.

Yield: 0.114 g, 44%, light-grey solids; Mp 189-191 °C (Lit. 207-208 °C)[57]; IR ν/cm^{-1} (ATR) 3178, 2021, 1629, 1566, 1493, 1444, 1092, 825; ^{1}H NMR (500 MHz, $CDCl_3$) δ/ppm 7.68-7.63 (2H, m, Ar-H), 7.51 (2H, d, J= 7.0 Hz, Ar-H, 7-H), 7.38 (3 H, m, Hz, 4-H, 5-H, 7-H), 7.21 (1H, t, *J*= 6.8 Hz, 6-H); ^{13}C NMR (125 MHz, $CDCl_3$) δ/ppm 163.4, 151.5, 138.4, 130.0, 129.5, 129.0, 126.3, 122.8, 121.1, 120.9, 119.7.

***N*-[4-(trifluoromethyl)phenyl]-1,3-benzothiazol-2-amine (5k)**

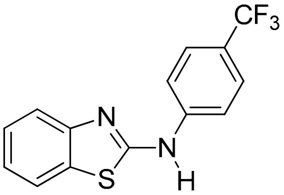

The product was purified by trituration of the crude in ethanol.

Yield: 0.09 g, 36 %, grey solids; Mp 173-175 °C (Lit. 194-196 °C)[57]; IR ν/cm^{-1} (ATR) 2963, 1594, 1519, 1315, 1105, 1063, 838; ^{1}H NMR (500 MHz, $CDCl_3$) δ/ppm 7.85-7.79 (2H, m, Ar-H), 7.65-7.56 (2H, m, Ar-H, 7-H), 7.30-7.25 (2 H, m, 5-H, 6-H); ^{13}C NMR (125 MHz, $CDCl_3$) δ/ppm 161.8, 151.7, 143.9, 132.6, 129.8, 127.9, 126.1, 124.9, 123.4, 122.2, 120.8, 118.2.

***N*-(1-phenylethyl)-1,3-benzothiazol-2-amine (5l)**

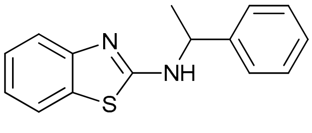

The product was purified by chromatography, hexane – EtOAc 4:1.

Yield: 0.18 g, 70%, waxy tan solids; (Lit. mp. 131-132 °C)[14]; IR ν/cm^{-1} (ATR) ^{1}H NMR (500 MHz, $CDCl_3$) δ/ppm 7.44-7.42 (2H, m, 5-H, 7-H), 7.38-7.36 (4H, m, 4-H, Ar-H), 7.32-7.28 (2H, m, Ar-H), 7.07 (1 H, t, *J* = 6.8 Hz, 6-H), 4.81 (1H, q, *J*= 6 Hz, CH), 1.64 (3H, d, *J*= 5.5 Hz, CH_3); ^{13}C NMR (125 MHz, $CDCl_3$) δ/ppm 167.5, 151.9, 143.0, 130.55, 128.8, 127.6, 126.2, 125.9, 121.4, 120.9, 118.8, 55.5, 23.8.

***N*-cyclohexyl-1,3-benzothiazol-2-amine (5m)**

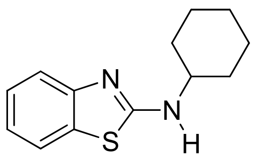

The product was purified by chromatography, hexane – EtOAc 5:1.

Yield: 0.10 g, 42%, viscous colourless oil, (Lit. mp. 78 -80 °C)[57]; IR ν/cm^{-1} (ATR) 2931, 2851, 1597, 1539, 1445, 1203, 1126; ^{1}H NMR (500 MHz, $CDCl_3$) δ/ppm 7.60 (1H, d, J = 8Hz, 7-H), 7.55 (1H, d, J = 8Hz, 4-H), 7.31 (1H, t, J = 7.5 Hz, 5-H), 7.09 (1H, t, J = 7.5 Hz, 6-H), 6.13 (1H, br s, NH), 3.40 - 3.60 (1H, m, NHC*H*), 2.16 - 2.00 (2H, m, CH_2), 1.80-1.60 (3H, m, CH_2, C*H*H), 1.44 – 1.22 (5H, m, CH_2 x 2, CH*H*); ^{13}C NMR (125 MHz, $CDCl_3$) δ/ppm 167.0, 152.5, 130.3, 125.9, 121.3, 120.8, 118.6, 54.8, 33.3, 25.5, 24.8.

2-(benzo[d]thiazol-2-ylamino)ethanol (5n)

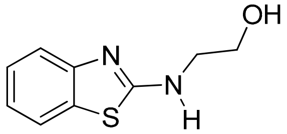

The product was purified by chromatography, hexane – EtOAc 4:1.

Yield: 0.08 g, 44%, tan solid, Mp 96.8-99.4 °C (Lit. 99 -101 °C)[28]; IR ν/cm^{-1} (ATR) 3194, 2958, 1595, 1542, 1142, 1216, 1019, 751; ^{1}H NMR (500 MHz, $CDCl_3$) δ/ppm 7.34 (m, 1H), 7.20 (m, 2H), 7.16 (m, 1H); ^{13}C NMR (125 MHz, $CDCl_3$) δ/ppm 167.6, 152.5, 130.3, 125.3, 120.8, 119.4,118.2, 60.86, 46.8.

***N*-phenylnaphtho[1,2-*d*][1,3]thiazol-2-amine (5q)**

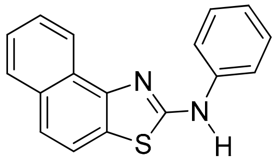

The product was purified by recrystallization from ethanol-water.

Yield: 0.26 g, 95%, grey solids; Mp 139.7-141.2 °C (Lit. 140 – 141 °C)[42]; IR ν/cm^{-1} (ATR) 3382, 3050, 1595, 1534, 1489, 1392. 902, 750; ^{1}H NMR (500 MHz, $CDCl_3$) δ/ppm 8.67 (1H, d, *J* = 7.8 Hz, 9-H), 7.95 (1H, d, *J* = 7.8 Hz, 4-H), 7.41 (1H, d, *J* = 8.5 Hz, 8-H), 7.68 (1H, d, *J* = 8.6 Hz), 7.61-7.50 (5H, m, Ar-H,), 7.43-7.40 (2H, m,)7.33 (1H, m,); ^{13}C NMR (125 MHz, $CDCl_3$) δ/ppm 164.1, 147.3, 140.1, 132.0, 129.5, 128.1, 126.2, 125.7, 124.0, 123.7, 122.9, 119.2, 118.7, 116.8.

Naphtho[1,2-*d*]thiazol-2-amine (5r)

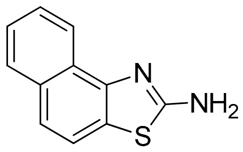

The product was purified by filtering through silica and eluting with hexane → hexane-EtOAc (2:1).

Yield: 0.06 g, 30%, tan solids; Mp 188.6-191.4 °C (Lit. 190-192 °C)[18]; IR ν/cm^{-1} (ATR) 3440, 3291, 3084, 1639, 1537, 1361, 1220, 1059, 795; ^{1}H NMR (500 MHz, $CDCl_3$) δ/ppm 8.47 (1H, d, J = 8.3, 9-H), 7.87 (1H, d, *J* = 8.5 Hz, 4-H), 7.66 (1H, d, *J* = 8.6 Hz, 8-H), 7.66 (1H, d, *J* = 8.6 Hz), 7.55 (1H, t, *J* = 7.6 Hz), 7.48 (1H, m), 5.59-5.55 (2H, br s, NH_2); ^{13}C NMR (125 MHz, $CDCl_3$) δ/ppm 166.4, 147.6, 132.1, 128.0, 126.9, 126.3, 126.1, 125.4, 123.7, 122.6, 118.7.

***N*-benzylnaphtho[1,2-*d*][1,3]thiazol-2-amine (5s)**

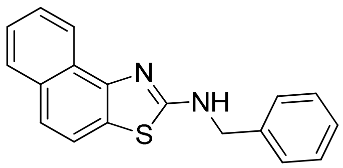

The product was purified by recrystallization from ethanol-water.

Yield: 0.27 g, 93%, white solids; Mp 107.3-109 °C (Lit. 104-106 °C)[18]; IR ν/cm^{-1} (ATR) 3210, 3028, 1590, 1557, 1353, 1194, 903, 794, ^{1}H NMR (500 MHz, $CDCl_3$) δ/ppm 8.56 (1H, d, *J* = 7.6 Hz, 9-H), 7.89 (1H, d, *J* = 7.8 Hz, 4-H), 7.69 (1H, d, *J* = 7.8 Hz, 8-H), 7.60-7.59 (1H, m, 7-H), 7.56-7.49 (2H, m, 5-H, 6-H), 7.45 (2H, m, Ar-H), 7.39-7.32 (3H, m, Ar-H), 6.42 (1H, br s, NH), 4.68 (2H, m, CH_2-Ar); ^{13}C NMR (125 MHz, $CDCl_3$) δ/ppm 168.3, 148.0, 137.5, 132.2, 128.8, 128.0, 127.8, 126.8, 125.9, 125.4, 125.1, 123.8, 122.3, 121.9, 118.8, 49.7.

***N*-cyclohexylnaphtho[1,2-*d*][1,3]thiazol-2-amine (5t)**

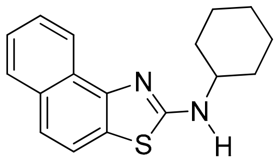

The product was purified by chromatography, hexane – EtOAc 5:1.

Yield: 0.25 g, 88%, light brown viscous oil; (Lit. mp. 115-116 °C)[42]; IR ν/cm^{-1} (ATR) 3388, 2927, 1537, 1364, 1072, 904, 798; ^{1}H NMR (500 MHz, $CDCl_3$) δ/ppm 8.52 (1H, d, *J* = 7.9 Hz, 9-H), 7.82 (1H, d, *J* = 8 Hz, 4-H), 7.63 (1H, d, *J* =8.5 Hz, 8-H), 7.51-7.47 (2H, m, 5-H, 6-H), 7.44-7.42 (1H, m, 7-H), 6.04 (1H, br s, NH), 3.45 (1H, m, CH-N), 2.10-2.08 (m, 2H, CHH), 1.71-1.69 (m, 2H, CHH), 1.58-1.56 (m, 1H, C*H*H), 1.38-1.31 (m, 3H, CHH), 1.26-1.20 (m, 2H, CHH); ^{13}C NMR (125 MHz, $CDCl_3$) δ/ppm. 167.8, 148.1, 132.2, 128.0, 126.7, 125.8, 125.3, 124.0, 121.4, 118.8, 55.2, 33.1, 25.5, 24.8.

***N,N*-dimethylnaphtho[1,2-d]thiazol-2-amine (5u)**

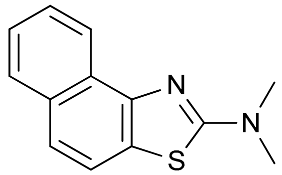

The product was purified by chromatography, hexane – EtOAc 4:1.

Yield: 0.10 g, 47%, light yellow solids; ; Mp 116.1-118.2 °C (Lit. 115-117 °C)[43]; IR (ATR) ν/cm^{-1} 2917, 1586, 1557, 1393, 1341, 935, 801; ^{1}H NMR (500 MHz, $CDCl_3$) δ/ppm 8.62 (1H, d, *J* = 8.1 Hz, 9-H), 7.88 (1H, d, *J* = 8.0 Hz, 4-H), 7.70 (1H, d, *J* =7.2 Hz, 8-H), 7.55-7.59 (2H, m, 5-H, 6-H), 7.50 (1H, t, *J* = 7.4 Hz, 7-H), 3.30 (6H, s, CH3 x 2); ^{13}C NMR (125 MHz, $CDCl_3$) δ/ppm 167.7, 147.4, 130.5, 126.2, 125.2, 124.0, 123.9, 122.4, 119.3, 117.1, 38.5.

2-(morpholin-4-yl)naphtho[1,2-*d*][1,3]thiazole (5v)

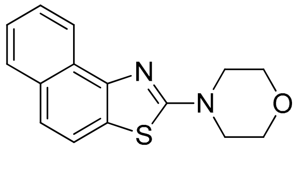

The product was purified by filtering through silica and eluting with hexane → hexane-EtOAc (2:1).

Yield: 0.23 g, 85%, tan solids; Mp 188.1-190 °C (Lit. 183-184 °C)[38]; IR (ATR) ν/cm^{-1} 2960, 1531, 1228, 1111, 913, 794; ^1H NMR (500 MHz, $CDCl_3$) δ/ppm 8.56 (1H, d, *J* = 8.0 Hz, 9-H), 7.86 (1H, d, *J* = 8.2 Hz, 4-H), 7.69 (1H, d, *J* = 8.6 Hz, 8-H), 7.58-7.54 (2H, m, 5-H, 6-H), 7.49-7.47 (1H, m, 7-H), 3.88-3.87 (4H, m, CH_2-N x 2), 3.70-3.69 (4H, m, CH_2-O x 2); ^{13}C NMR (125 MHz, $CDCl_3$) δ/ppm 169.5, 148.3, 132.2, 127.9, 127.3, 127.1, 125.9, 123.9, 121.9, 118.6, 66.3, 48.6.

2-(pyrrolidin-1-yl)naphtho[1,2-*d*][1,3]thiazole (5w)

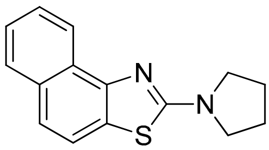

The product was purified by filtering through silica and eluting with hexane → hexane-EtOAc (2:1).

Yield: 0.21 g, 83%, off-white solids; Mp 125-127 °C (Lit. 143-145 °C)[38]; IR (ATR) ν/cm^{-1} 3050, 2954, 1584, 1547, 1356, 1305, 943, 798; ^1H NMR (500 MHz, $CDCl_3$) δ/ppm 8.64 (1H, d, *J* = 8 Hz, 9-H), 7.88 (1H, d, *J* = 8.2 Hz, 4-H), 7.71 (1H, d, *J* = 8.5 Hz, 8-H), 7.61-7.50 (3H, m, 5-H, 6-H, 7-H), 3.66 (4H, m, CH_2-N x 2), 2.11 (4H, m, CH_2-CH_2); ^{13}C NMR (125 MHz, $CDCl_3$) δ/ppm 166.0, 149.2, 132.2, 127.9, 126.8, 126.3, 125.6, 125.1, 124.1, 120.7, 118.8, 49.6, 25.7.

2-phenyl-6-methylbenzothiazole (6b)

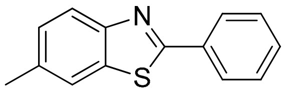

The product was purified by chromatography, hexane – EtOAc 9:1.

Yield = 0.006 g, 5% (Method E), white solids; Mp 120.1-122 °C (Lit 118-119 °C)[30]; IR (ATR) ν/cm^{-1} 1590, 1524, 1505, 1405, 1334, 1221, 1101, 821, 731;^1H NMR (200 MHz, $CDCl_3$) δ/ppm 8.00 - 7.95 (2H, m, 2'-H, 6'-H), 7.86 (1H, d, *J* =8.4 Hz, 4-H), 7.56 (1H, -s, 7-H), 7.40 – 7.37 (3H, m, 3' - 5'-H), 7.20 (1H, d, *J* = 8.4 Hz, 5-H), 2.39 (3H, s, CH_3), ^{13}C NMR (50 MHz, $CDCl_3$) δ/ppm 166.9, 152.4, 135.3, 133.9, 130.6, 128.9, 127.8, 127.4, 122.8, 121.3, 21.4.

2-phenyl-6-methoxybenzothiazole (6c)

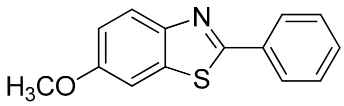

The product was purified by chromatography, hexane – EtOAc 5:1

Yield = 0.01 g, 11 % (Method E), white solids; Mp 113.1- 115.4 °C (Lit. 114-116 °C)[30]; IR (ATR) ν/cm^{-1} 1602, 1512, 1483, 1463, 1435, 1265, 1226, 1060, 1025, 970, 893, 823, 761; ^1H NMR (500 MHz, $CDCl_3$) δ/ppm 8.08 – 8.07 (2H, m, 2'-H, 6'-H), 7.99 (1H, d, *J*= 9 Hz, 4-H), 7.51-7.50 (3H, m, 3' - 5'-H), 7.39 (1H, s, 7-H), 7.13 (1H, d, *J* = 8.5 Hz, 5-H), 3.93 (3H, s, OCH_3); ^{13}C NMR (125 MHz, $CDCl_3$) δ/ppm 165.6, 157.8, 148.6, 136.4, 133.7, 130.6, 129.0, 127.3, 123.7, 115.7, 104.2, 55.8.

2-phenyl-4-methoxybenzothiazole (6d)

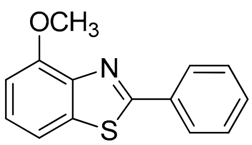

The product was purified by chromatography, hexane – EtOAc 1:1.

Yield = 0.05 g, 17 % (Method E), white solids; Mp 100.1 -102.8 °C (Lit. 99 – 100 °C)[27]; IR (ATR) ν/cm-1; 1H NMR (500 MHz, $CDCl_3$) δ/ppm 8.14 – 8.15 (2H, m, 2'-H,6'-H), 7.49 – 7.52 (4H, m, 6-H, 3' - 5'-H), 7.36 (1H, t, J = 8, 5-H), 6.95 (1H, d, J = 8, 7-H), 4.11 (3H, s, OCH_3); 13C NMR (125 MHz, $CDCl_3$) δ/ppm 166.8, 153.6, 144.3, 136.7, 133.5, 130.8, 128.9, 127.0, 126.2, 113.7, 106.8, 56.1.

4,7-Dimethoxy-2-phenylbenzothiazole (6e)

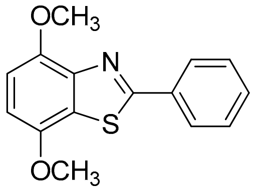

The product was purified by chromatography, hexane – CH_2Cl_2, 1:1

Yield: 0.02 g, 16% (Method E at ambient temperature),

0.03 g, 24% (Method E at 60 °C).

White solids; Mp 119.2 – 121.5 °C (Lit. 122-124 °C)[30]; IR ν/cm-1 (ATR) 1502, 1460, 1267, 1217, 1054, 794, 767, 658; 1H NMR (500 MHz, $CDCl_3$) δ/ppm 8.17 (2H, m, H-2', 6'), 7.50 -7.48 (3H, m, 3'-5'-H), 6.87 (1H, d, *J* = 9 Hz, 5-H), 6.78 (1H, d, *J* = 9 Hz, 6-H), 4.13 (3H, s, OCH_3), 4.02 (3H, s, OCH_3); 13C NMR (125 MHz, $CDCl_3$) δ/ppm 168.0, 148.5, 145.7, 133.9, 131.2, 129.2, 128.0, 125.9, 107.5, 105.7, 56.8, 56.4.

2-phenyl-5-methoxybenzothiazole (6f)

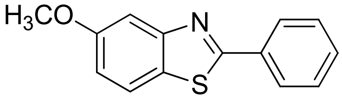

The product was purified by chromatography, hexane – EtOAc 1:1.

Yield = 0.13 g, 57 % (Method E), white solids; Mp 74.7-76.4 °C (Lit. 75 – 77 °C)[30]; IR (ATR) ν/cm-1 3000, 1600, 1561, 1461, 1426, 1329, 1280, 1248, 1161, 1076, 1025, 970; 1H NMR (500 MHz, $CDCl_3$) δ/ppm 8.08 (2H, m, 2'-H, 6'-H), 7.68 (1H, d, J = 8.5 Hz), 7.59 (1H, s, 4-H), 7.46 (3H, m, 3'- 5'-H), 7.02 (1H, d, J = 8.5 Hz, 5-H), 3.87 (3H, s, OCH_3); 13C NMR (125 MHz, $CDCl_3$) δ/ppm 169.2, 159.1, 155.4, 133.7, 130.9, 129.0, 127.2, 121.8, 115.4, 105.5, 55.5.

***N*-(morpholino(phenylimino)methyl)-N-phenylmorpholine-4-carbothioamide (7)**

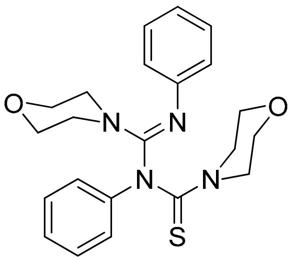

The product was purified by chromatography, hexane – EtOAc 1:1.

Yield: 0.14 g, 70% (Method E),

0.19 g, 90% (Method F)

White solids; Mp. 159 - 161 °C (Lit. 163-165 °C)[38]; IR ν/cm-1 (ATR) 1633, 1586, 1483, 1421, 1299, 1232, 1109, 1062, 1023; 1H NMR (500 MHz, $CDCl_3$) δ/ppm 7.49 – 7.27 (2H, m, Ar-H), 7.26-7.17 (2H, m, Ar-H), 7.17 – 7.11 (1H, m, Ar-H), 7.11– 6.94 (4H, m, Ar-H), 6.88 – 6.73 (1H, br s, Ar-H), 3.88 – 2.48 (16H, m, CH_2-O x 4, CH_2-N x 4), 13C NMR (125 MHz, $CDCl_3$) δ/ppm 185.1, 149.2, 142.7, 129.7, 128.7, 124.7, 122.7, 122.1, 122.0, 66.2, 65.4, 50.6, 46.7.

Table S1: Attempts towards the *N*-halosuccinimide mediated cyclization of thiobenzanilides[a]

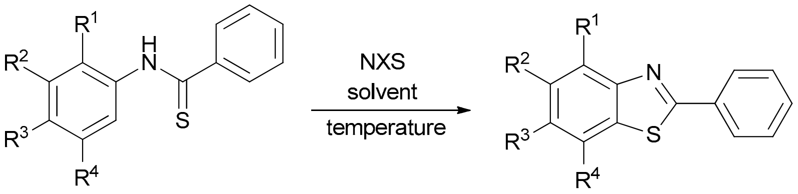

4a-f 6a-f

entry	substrate	R¹	R²	R³	R⁴	NXS	X ⁻	solvent	temperature	% yield[b]
1	8a	H	H	H	H	NBS	Bu_4NBr	DME	rt	-[c]
2	8a	H	H	H	H	NBS	Bu_4NBr	DME	100 °C	-[d]
3	8a	H	H	H	H	NBS	Bu_4NBr	AcOH	rt	-[c]
4	8a	H	H	H	H	NBS	-	CCl_4:CH_2Cl_2 (1:1)	rt	-[e]
5	8a	H	H	H	H	NCS	-	CCl_4:CH_2Cl_2 (1:1)	rt	-[e]
6	8a	H	H	H	H	NIS	-	CCl_4:CH_2Cl_2 (1:1)	rt	-[e]
7	8a	H	H	H	H	NBS	-	TFE	rt -60 °C	-[e]
8	8a	H	H	H	H	NBS	-	MeCN	rt -60 °C	-[e]
9	8a	H	H	H	H	NBS	-	DME	rt -60 °C	-[e]
10	8b	H	H	CH_3	H	NBS	-	DME	rt	-[e]
11	8c	H	H	OCH_3	H	NBS	-	DME	rt	-[e]
12	8b	H	H	CH_3	H	NBS	Bu_4NBr	DME	rt	5[f]
13	8c	H	H	OCH_3	H	NBS	Bu_4NBr	DME	rt	11[f]
14	8d	OCH_3	H	H	H	NBS	Bu_4NBr	DME	rt	17[f]
15	8e	OCH_3	H	H	OCH_3	NBS	Bu_4NBr	DME	rt	16[f]
16	8e	OCH_3	H	H	OCH_3	NBS	Bu_4NBr	DME	60 °C	24[f]
17	8f	H	OCH_3	H	H	NBS	Bu_4NBr	DME	rt	57[f]

[a] Reactions were carried out on a 1 mmol scale, Bu_4NBr (1 equiv), NBS (1 equiv.) in solvent (4 mL) for 24 hours. [b] Isolated yield. [c] A mixture of starting material and the corresponding benzamide was obtained. [d] Benzamide formation and ring halogenation occurred. [e]Complete conversion to benzamide. [f] The corresponding benzamide was also obtained in 16 – 44% yield.

^{1}H NMR (200 MHz, $CDCl_3$) of compound **5a**

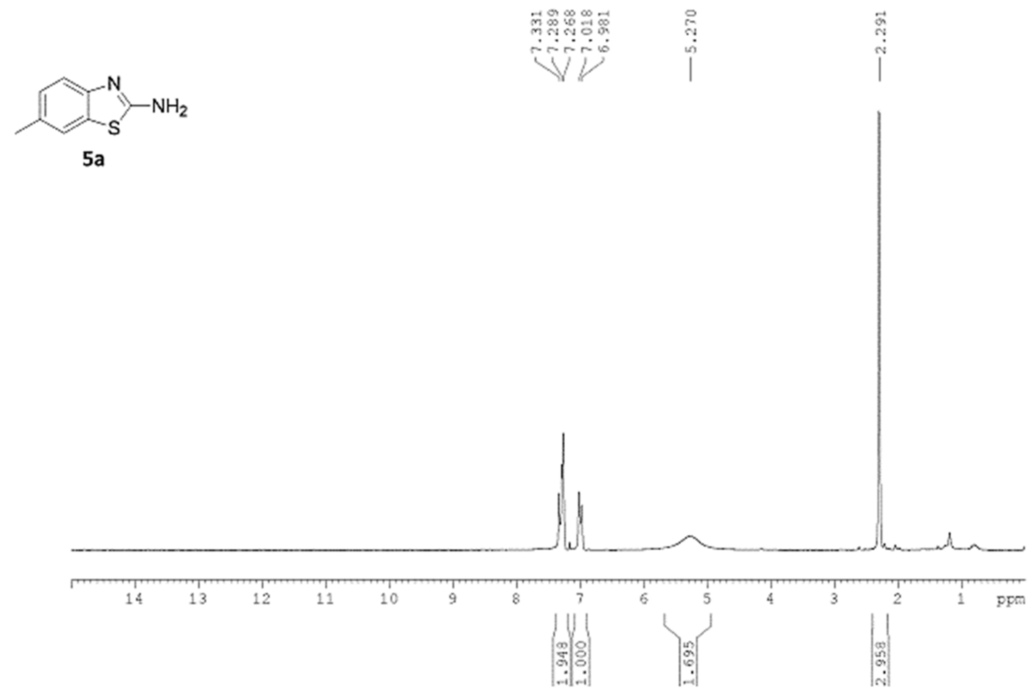

^{13}C NMR (50 MHz, $CDCl_3$) of compound **5a**

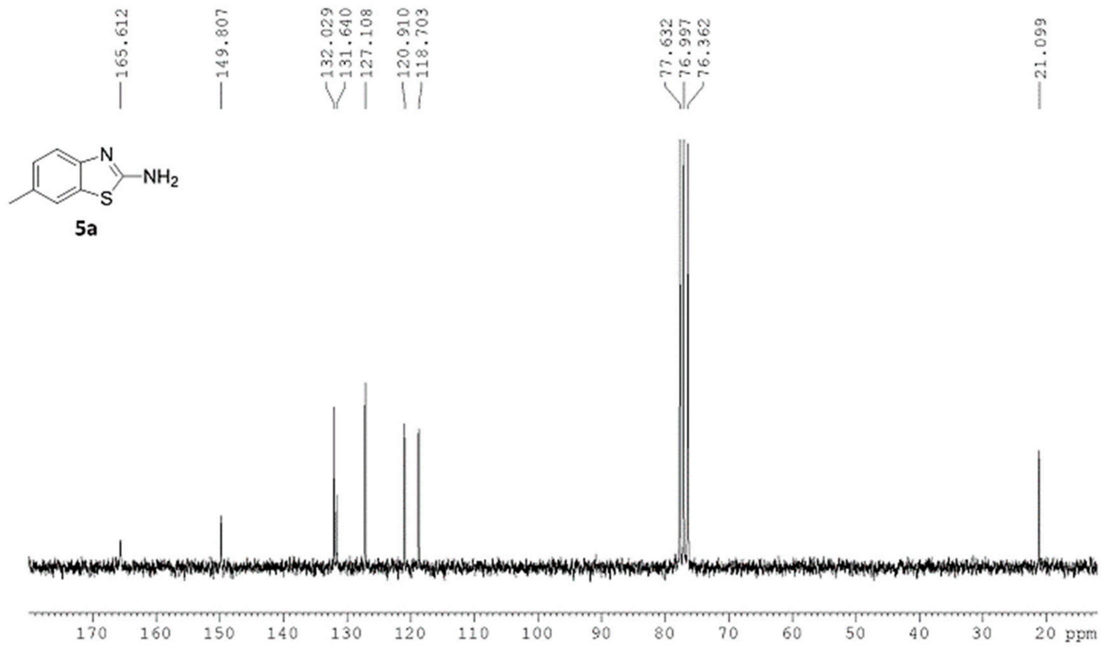

^{1}H NMR (500 MHz, DMSO-d_6) of compound **5b**

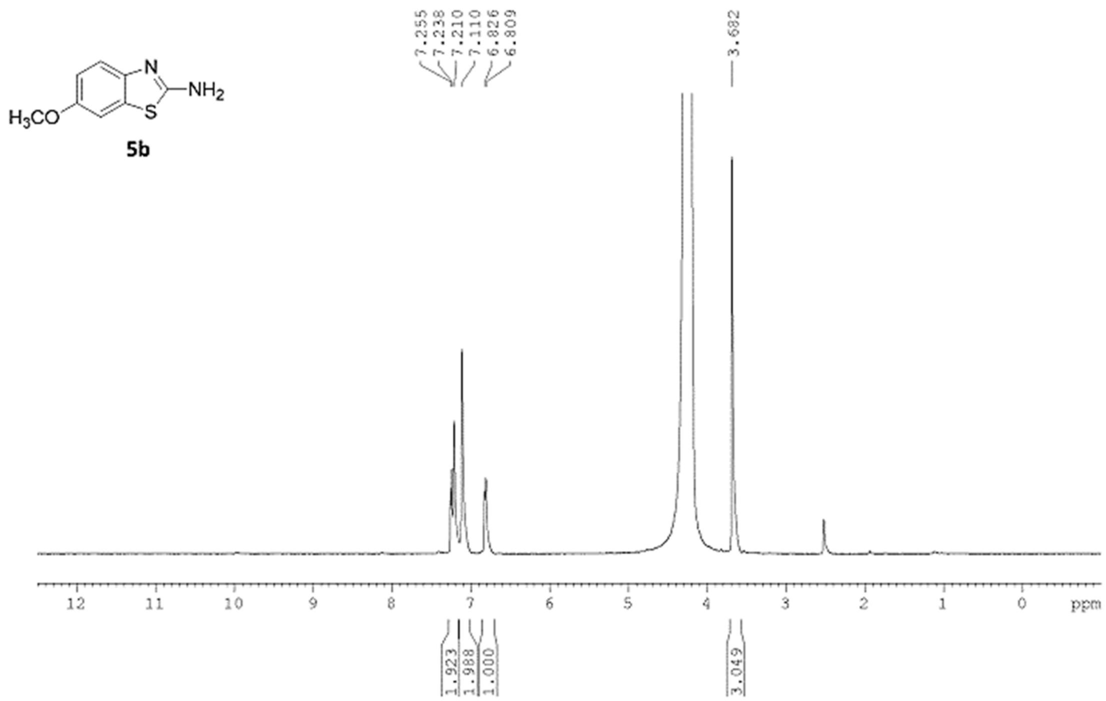

^{13}C NMR (125 MHz, DMSO-d$_6$) of compound **5b**

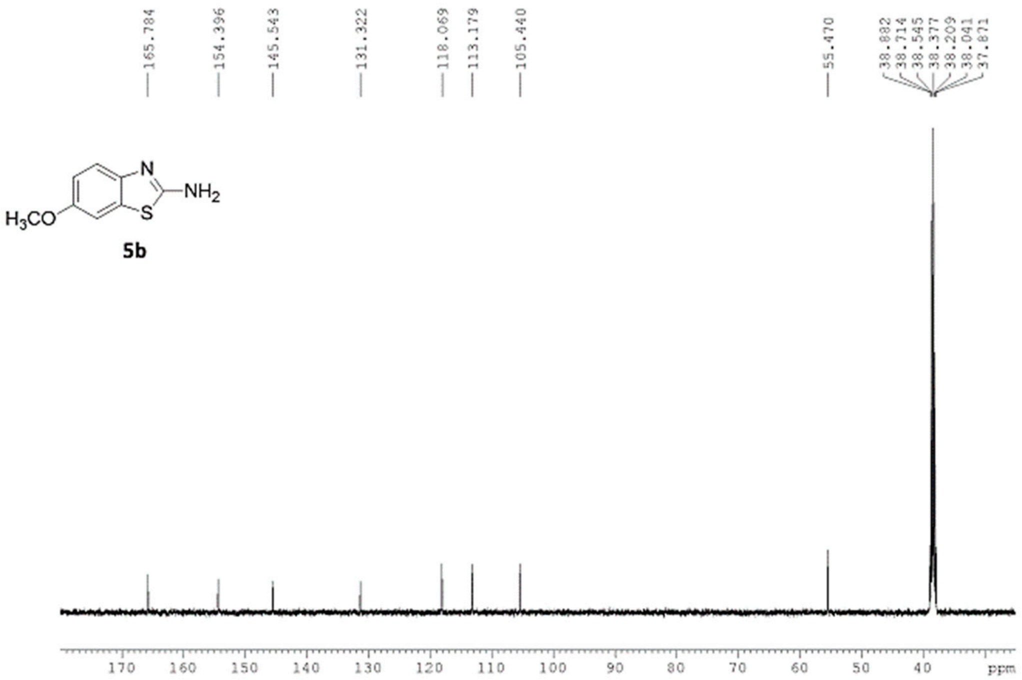

^{1}H NMR (500 MHz, acetone-d$_6$) of compound **5c**

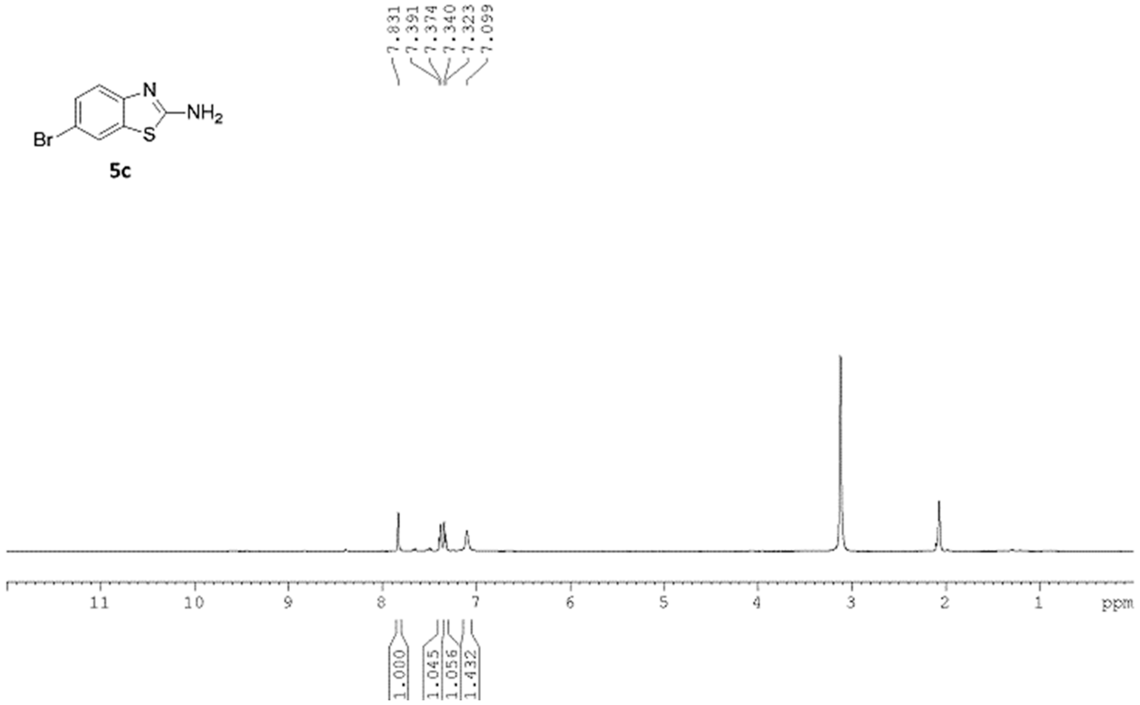

^{13}C NMR, APT (125 MHz, acetone-d$_6$) of compound **5c**

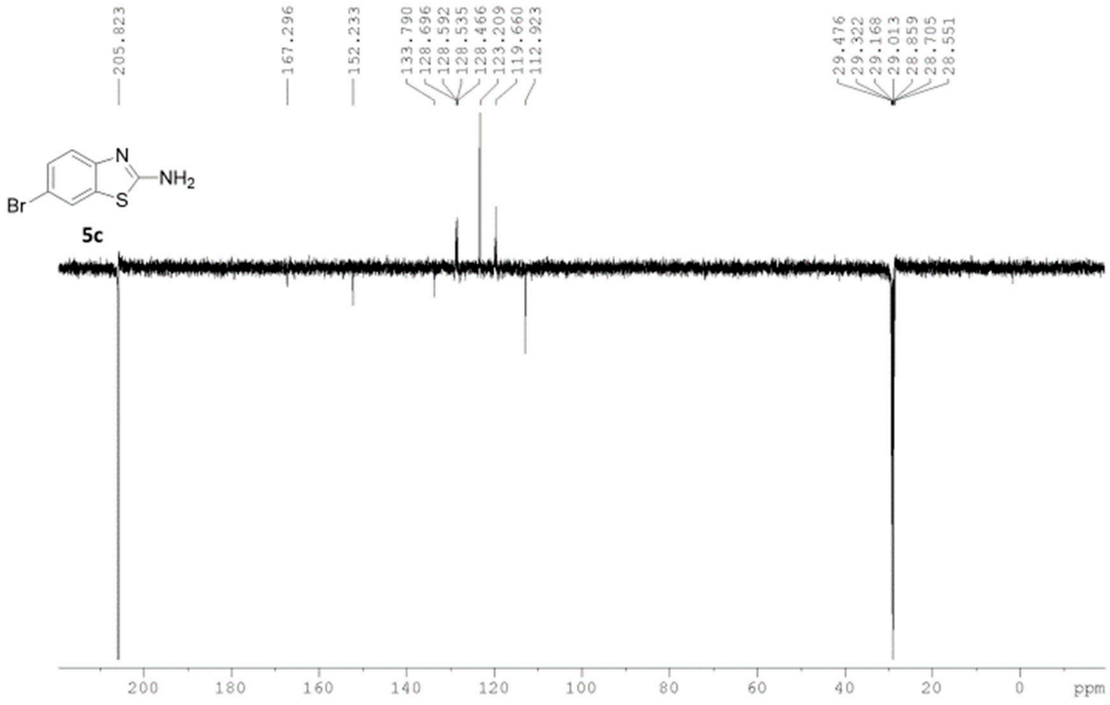

^{1}H NMR (500 MHz, $CDCl_3$) of compound **5e**

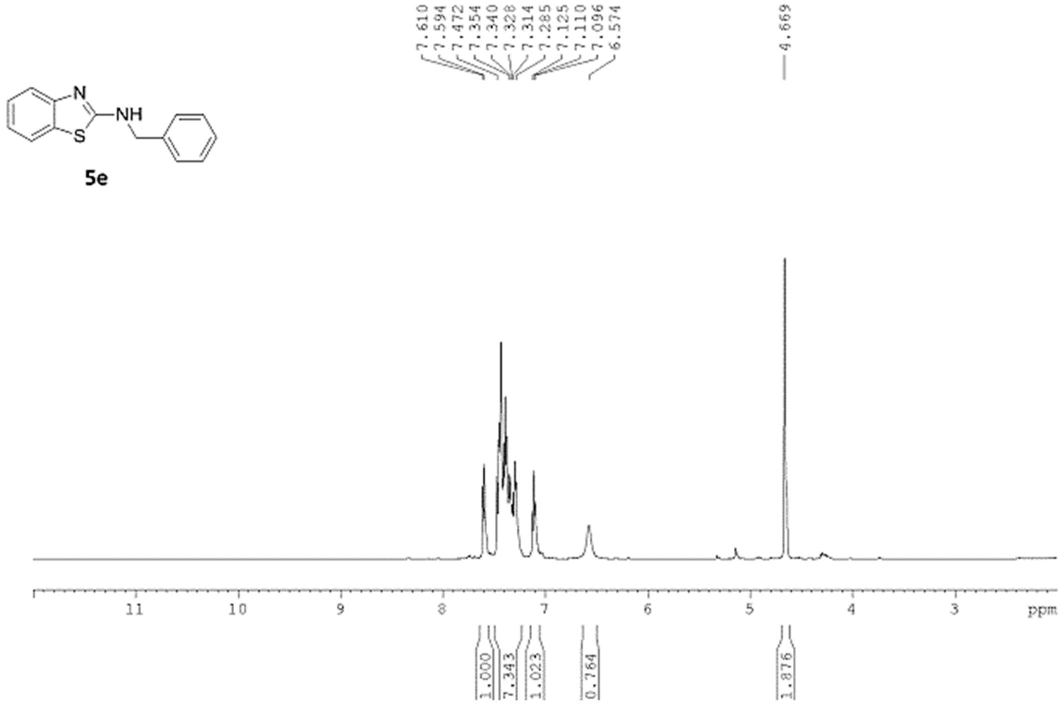

^{13}C NMR, APT (125 MHz, $CDCl_3$) of compound **5e**

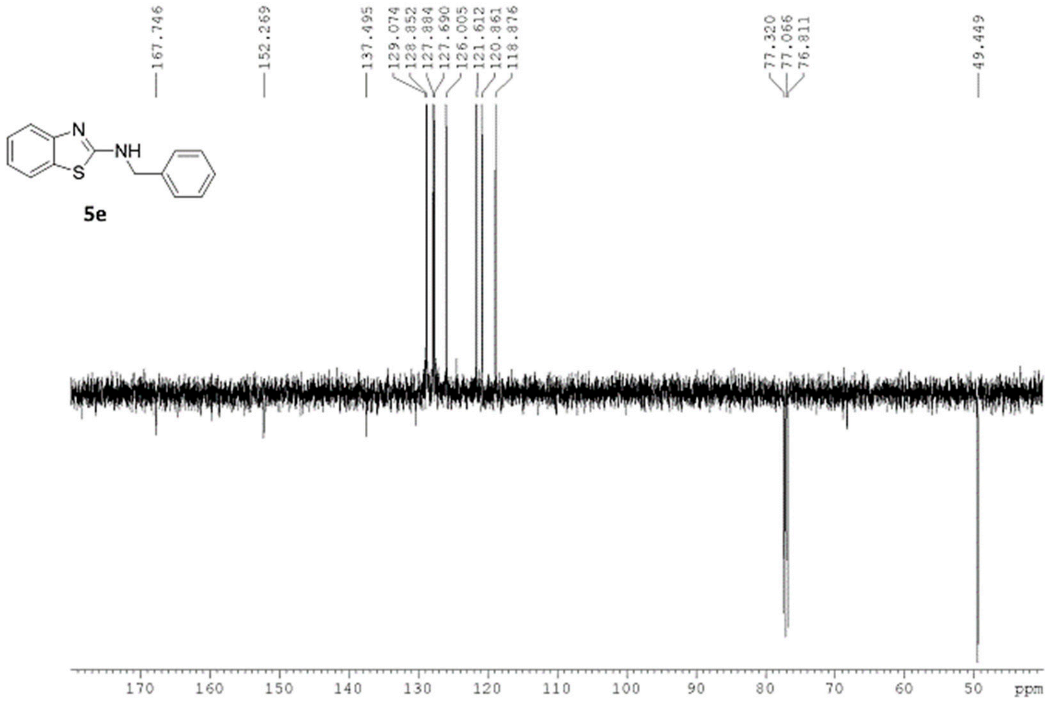

^{1}H NMR (200 MHz, $CDCl_3$) of compound **5f**

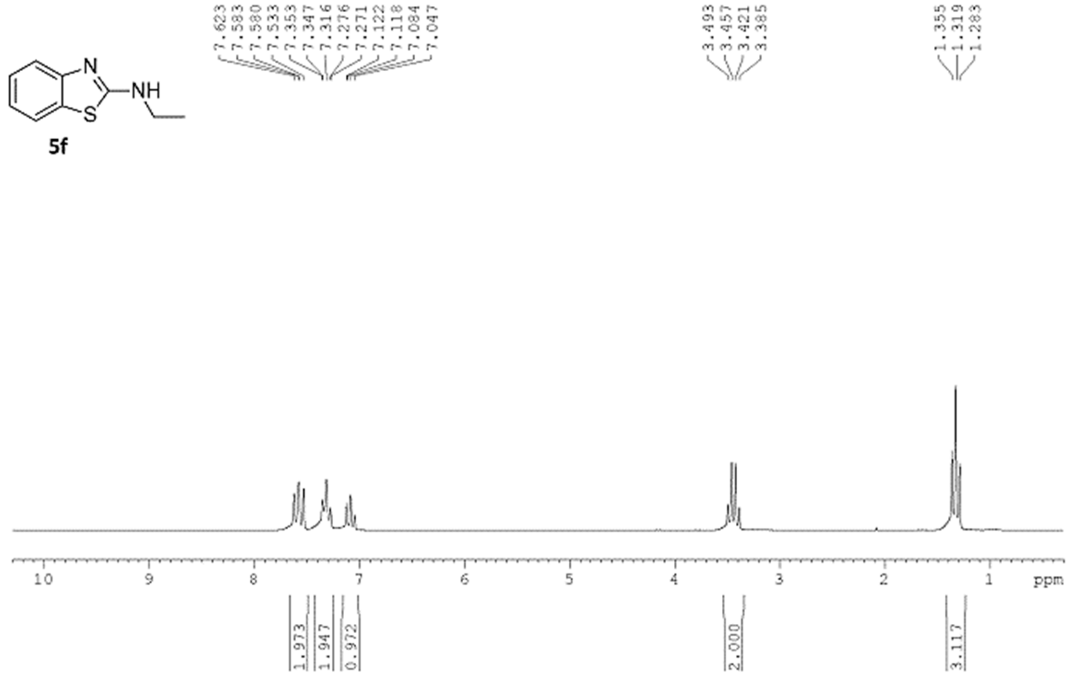

^{13}C NMR (50 MHz, $CDCl_3$) of compound **5f**

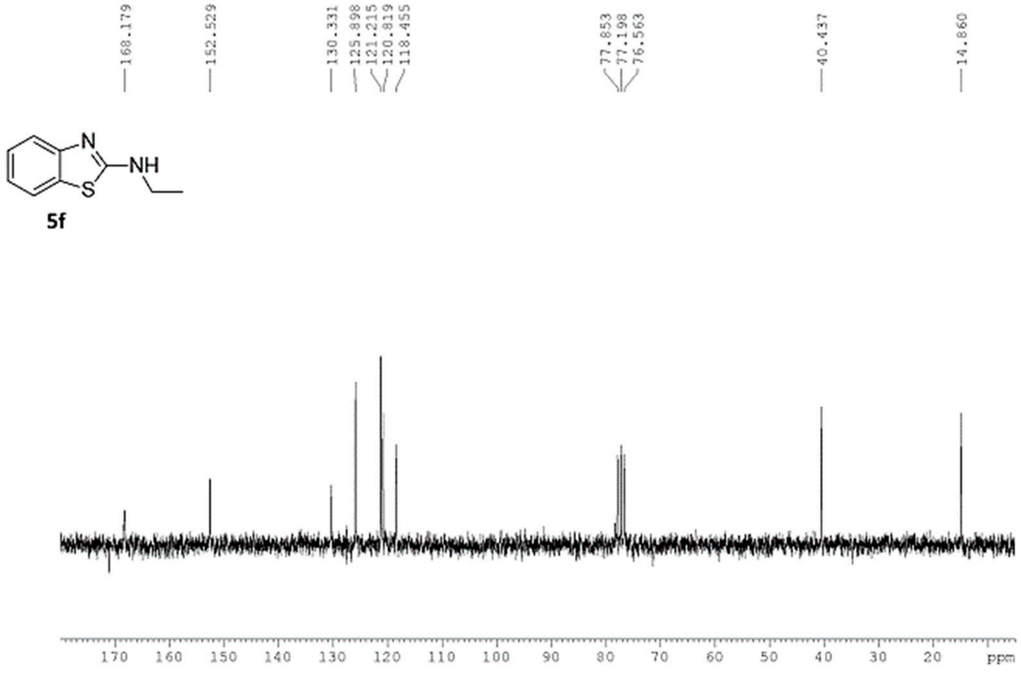

^{1}H NMR (500 MHz, acetone-d6) of compound **5g**

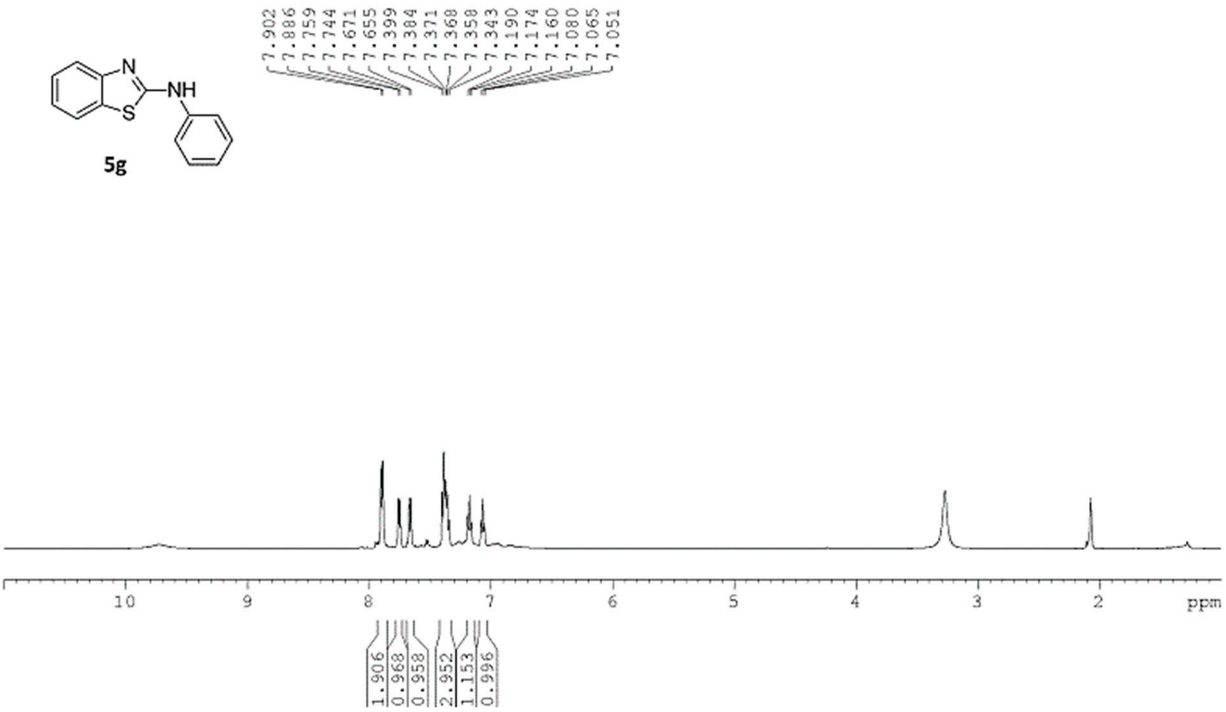

^{13}C NMR, APT (125 MHz, acetone-d6) of compound **5g**

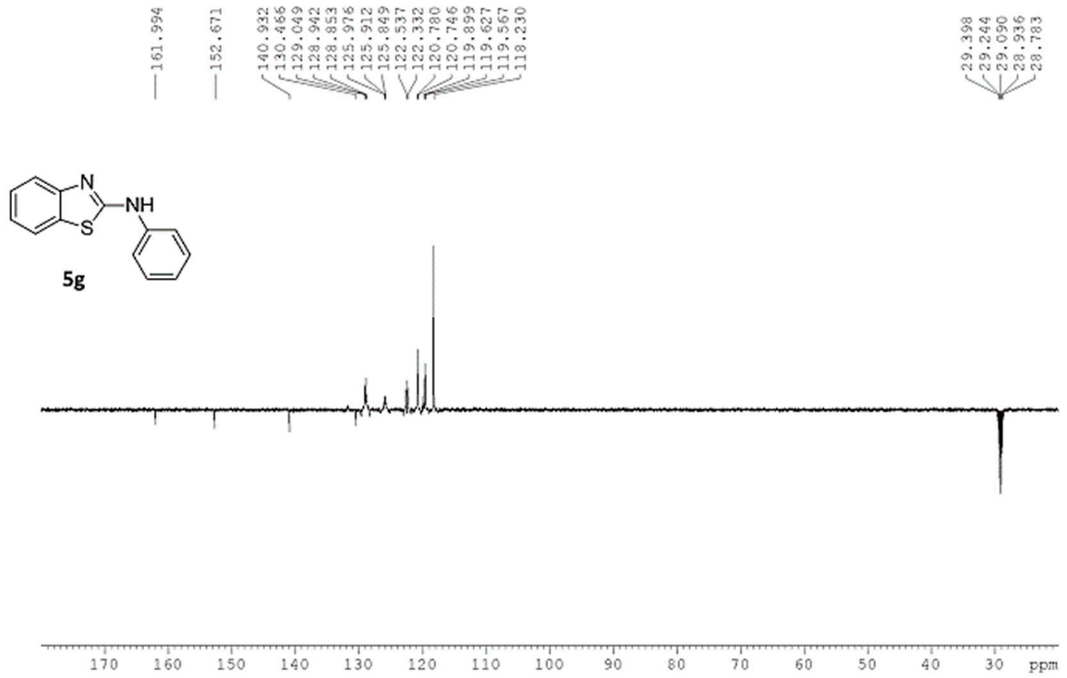

^{1}H NMR (500 MHz, $CDCl_3$) of compound **5j**

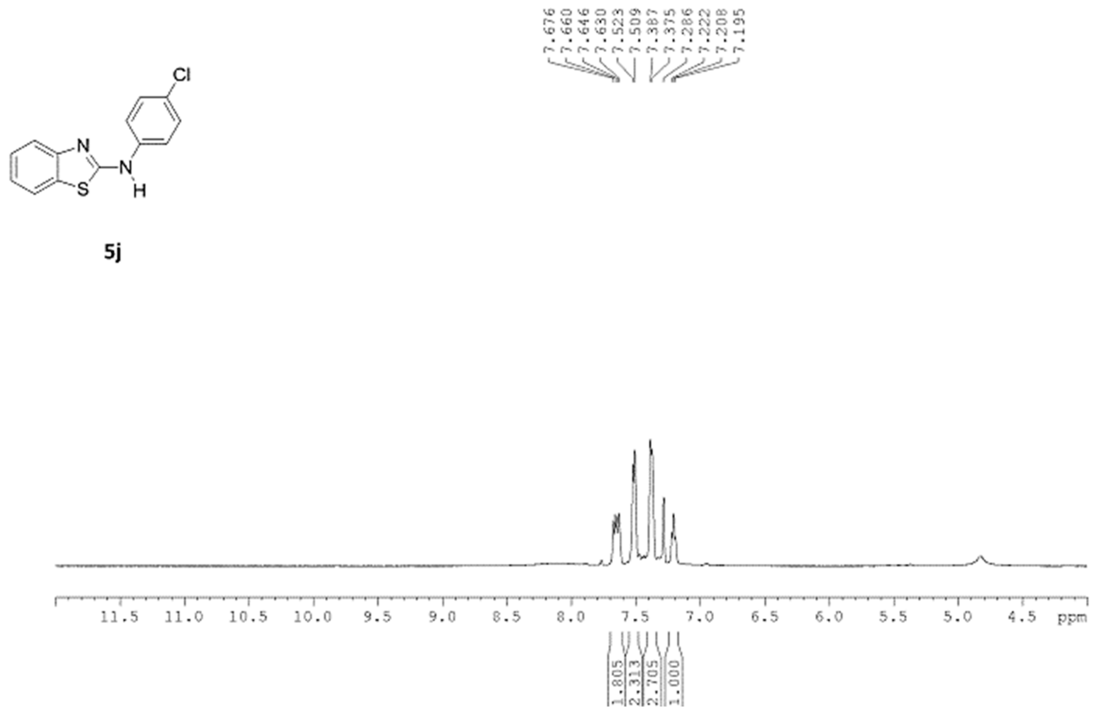

^{13}C NMR (125 MHz, $CDCl_3$) of compound **5j**

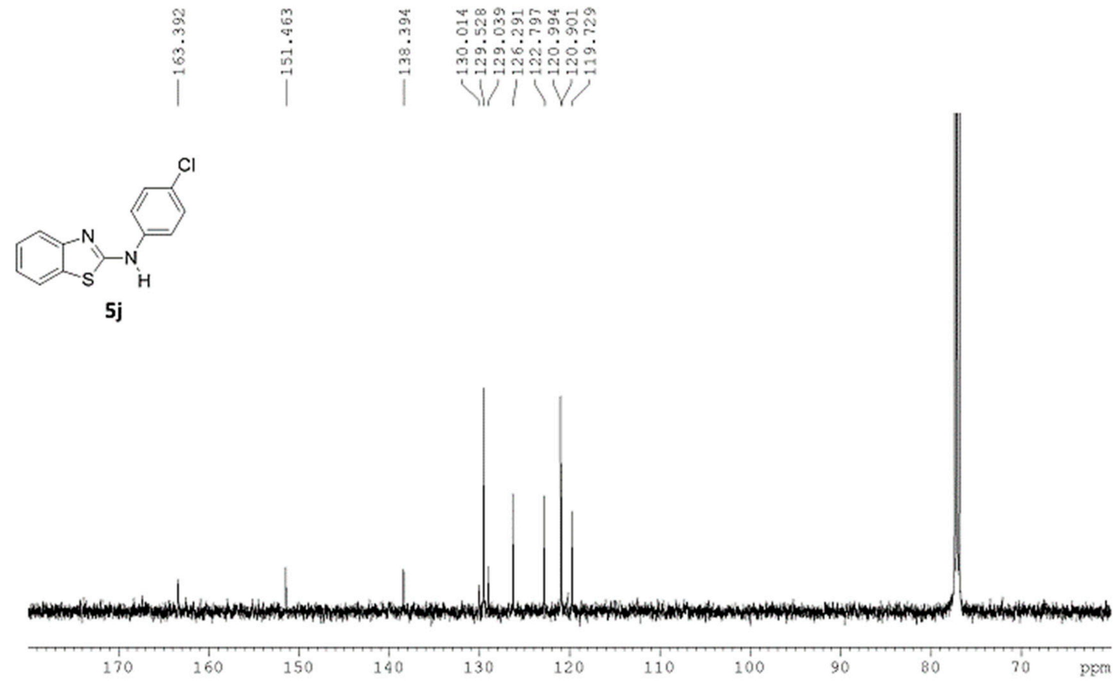

^{1}H NMR (500 MHz, $CDCl_3$) of compound **5m**

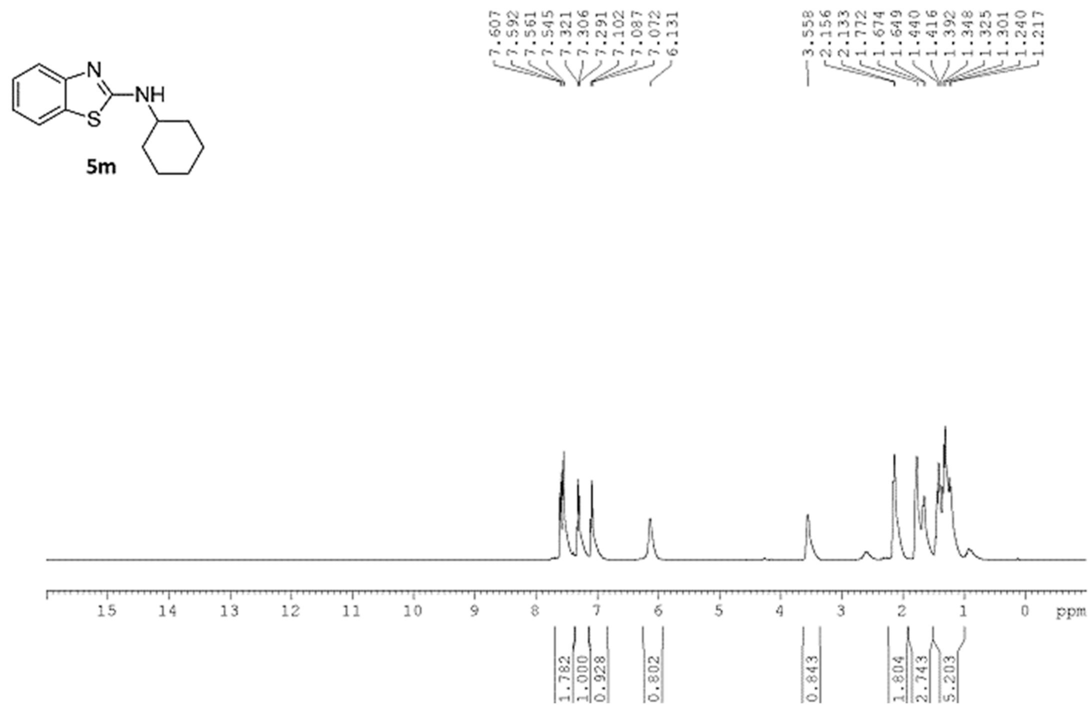

^{13}C NMR, APT (125 MHz, $CDCl_3$) of compound **5m**

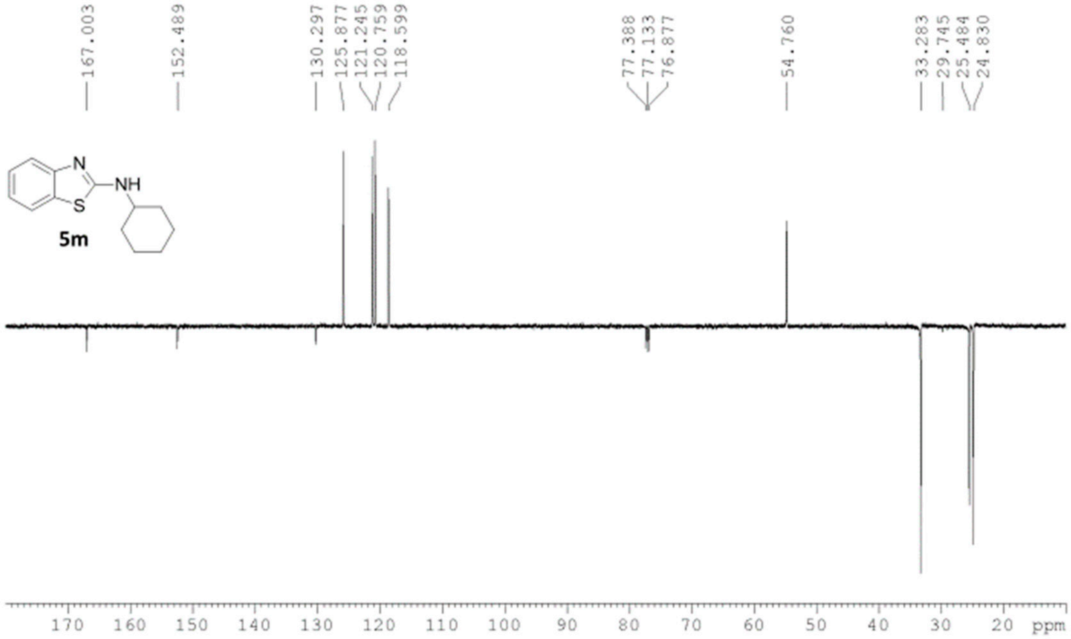

^{1}H NMR (500 MHz, $CDCl_3$) of compound **5r**

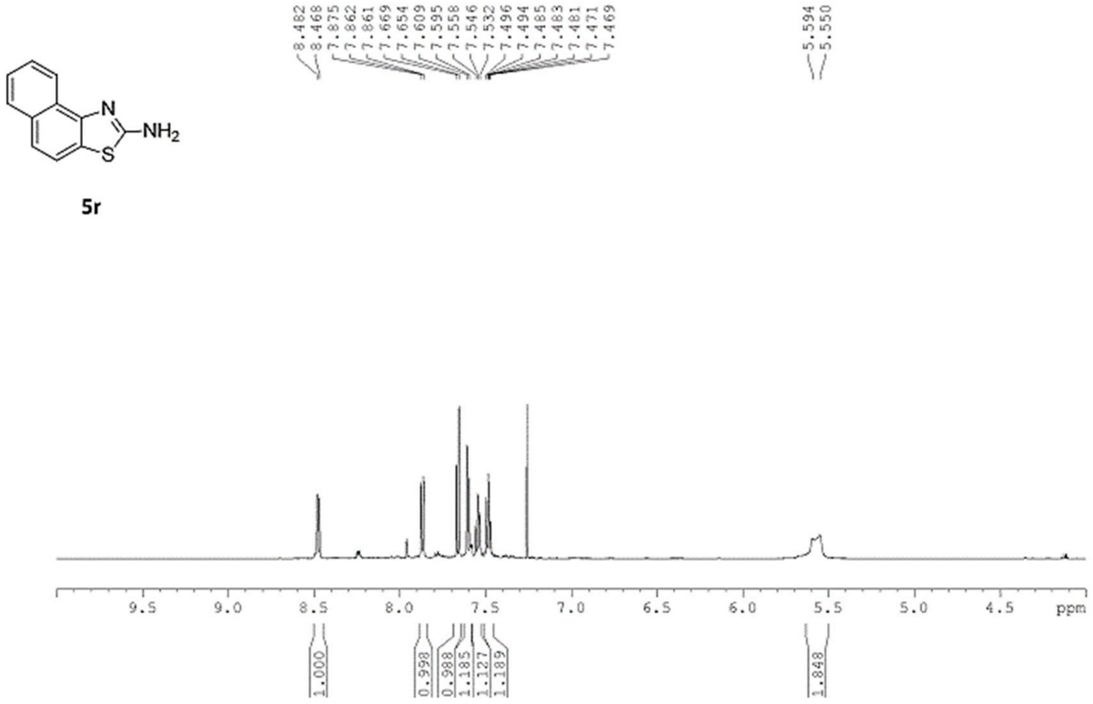

^{13}C NMR (125 MHz, $CDCl_3$) of compound **5r**

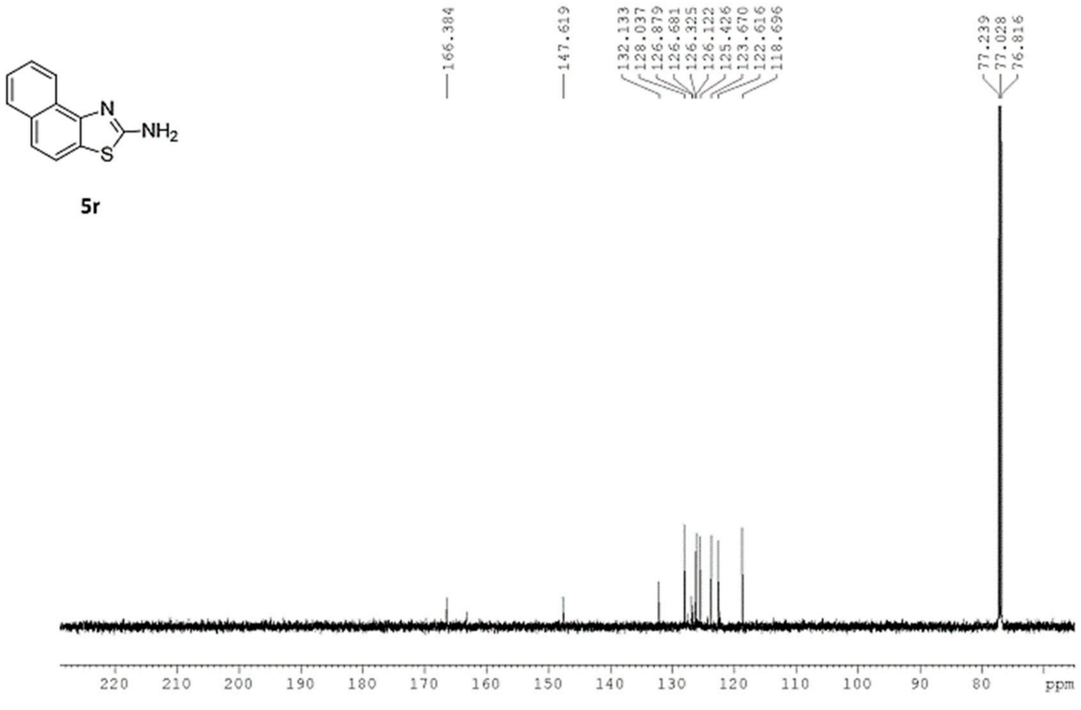

^{1}H NMR (500 MHz, $CDCl_3$) of compound **5s**

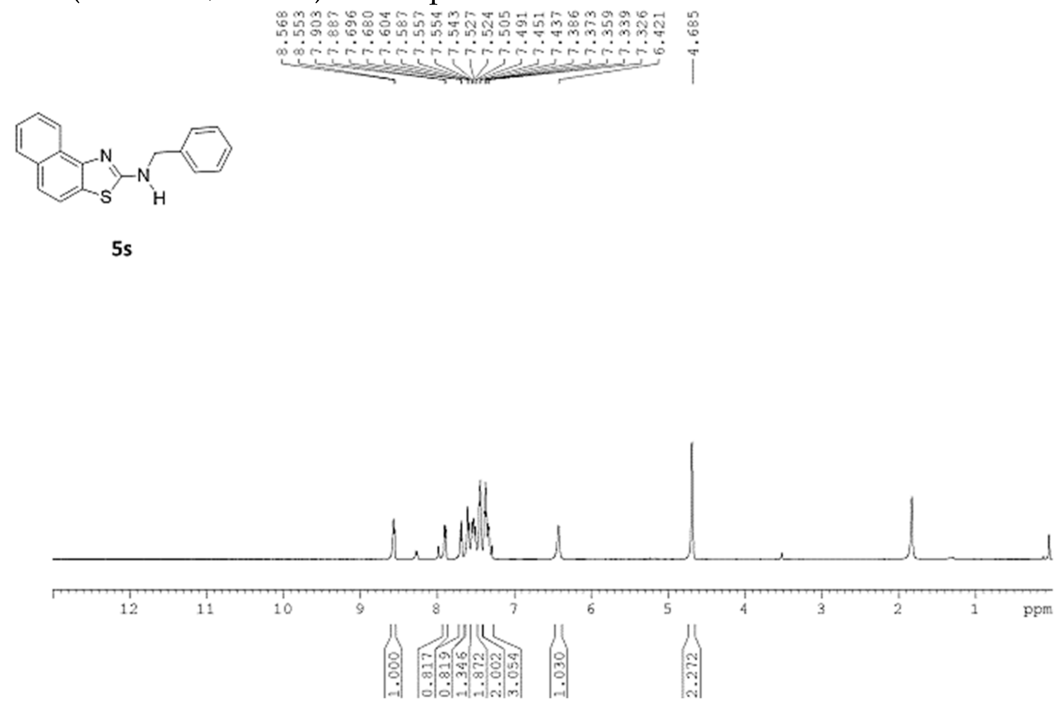

^{13}C NMR (125 MHz, $CDCl_3$) of compound **5s**

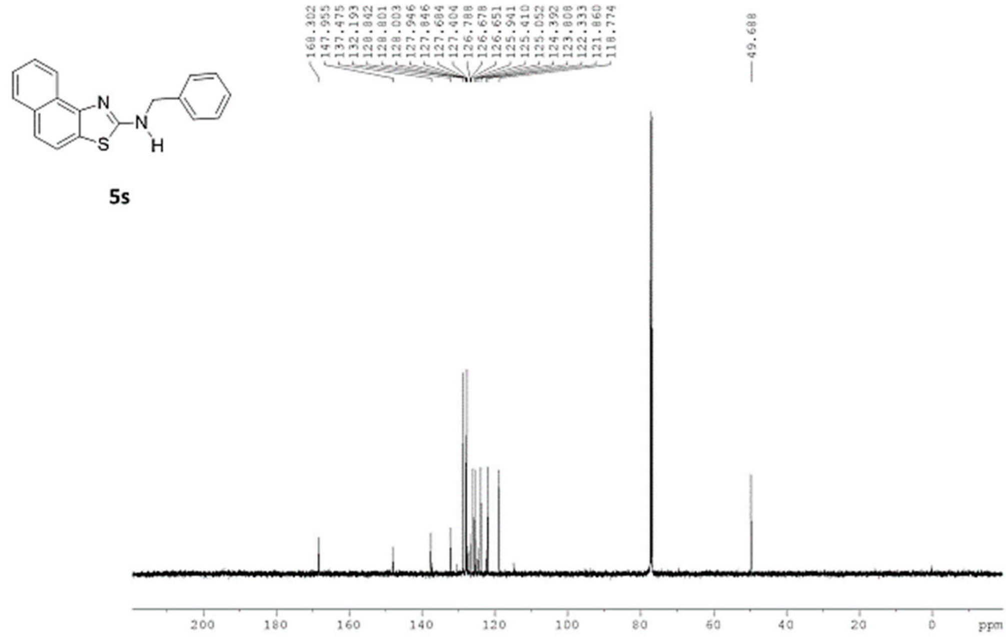

^{1}H NMR (500 MHz, $CDCl_3$) of compound **5t**

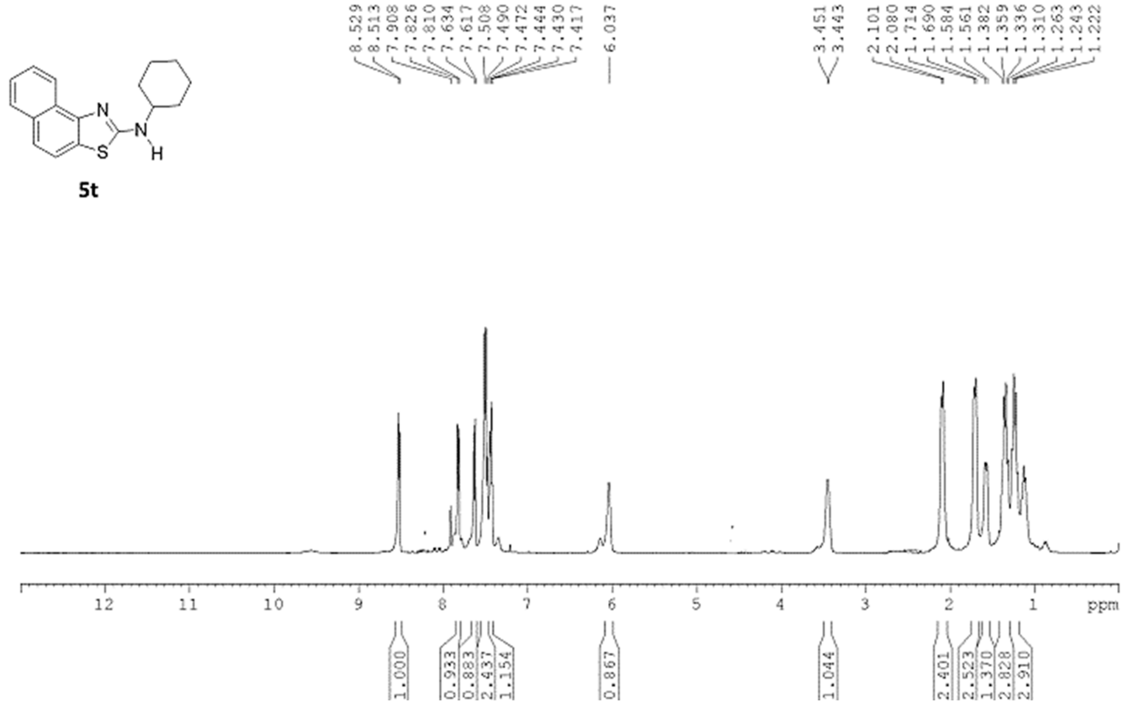

^{13}C NMR (125 MHz, $CDCl_3$) of compound **5t**

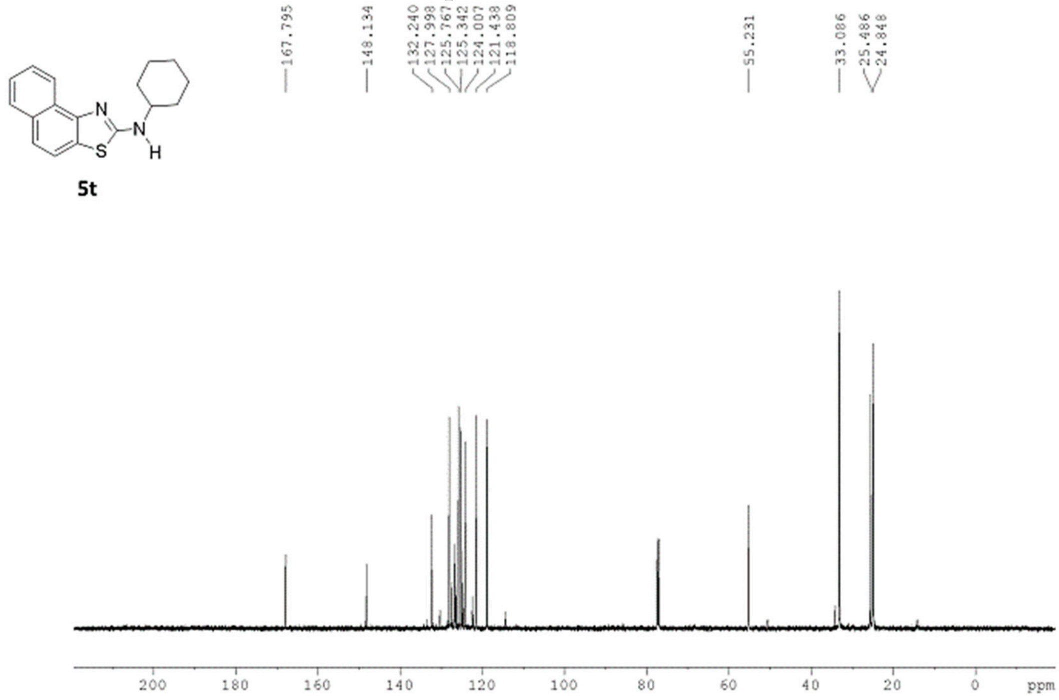

^{1}H NMR (500 MHz, $CDCl_3$) of compound **5u**

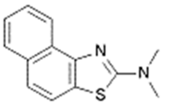

5u

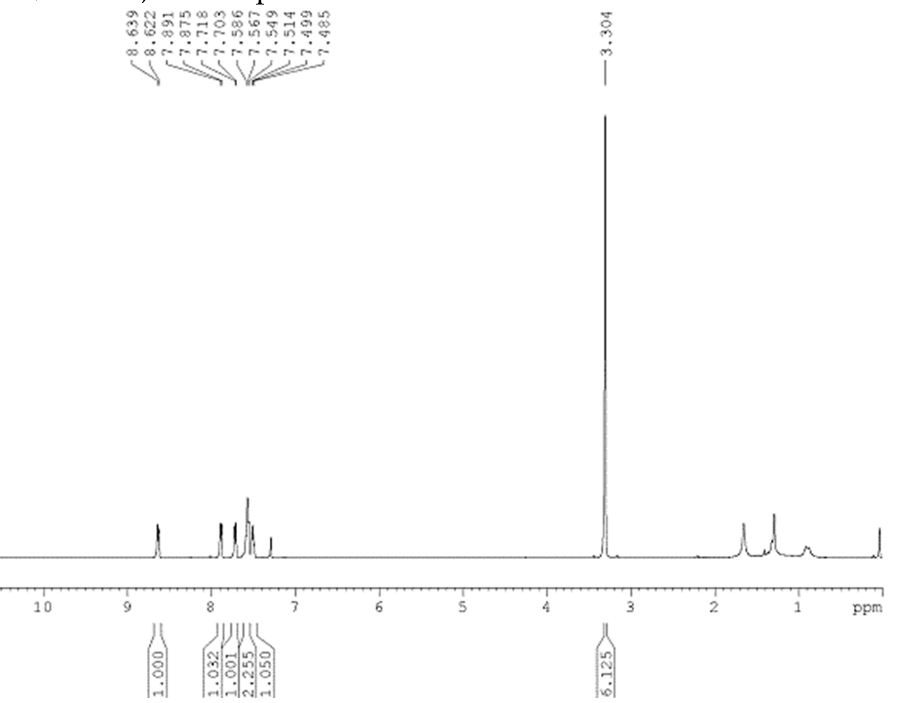

^{13}C NMR (125 MHz, $CDCl_3$) of compound **5u**

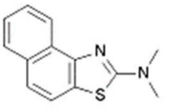

5u

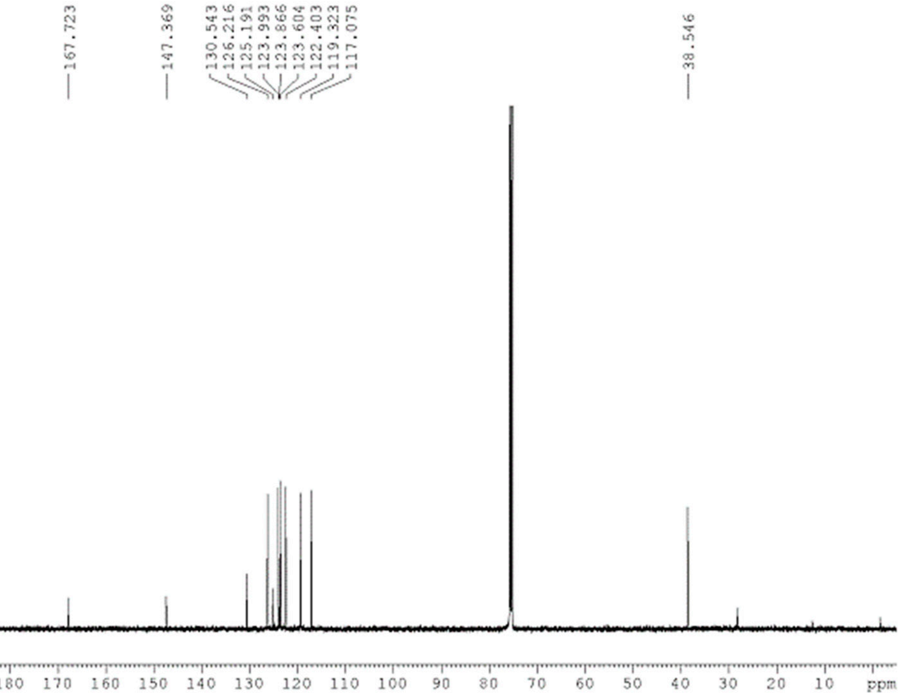

^{1}H NMR (500 MHz, $CDCl_3$) of compound **5v**

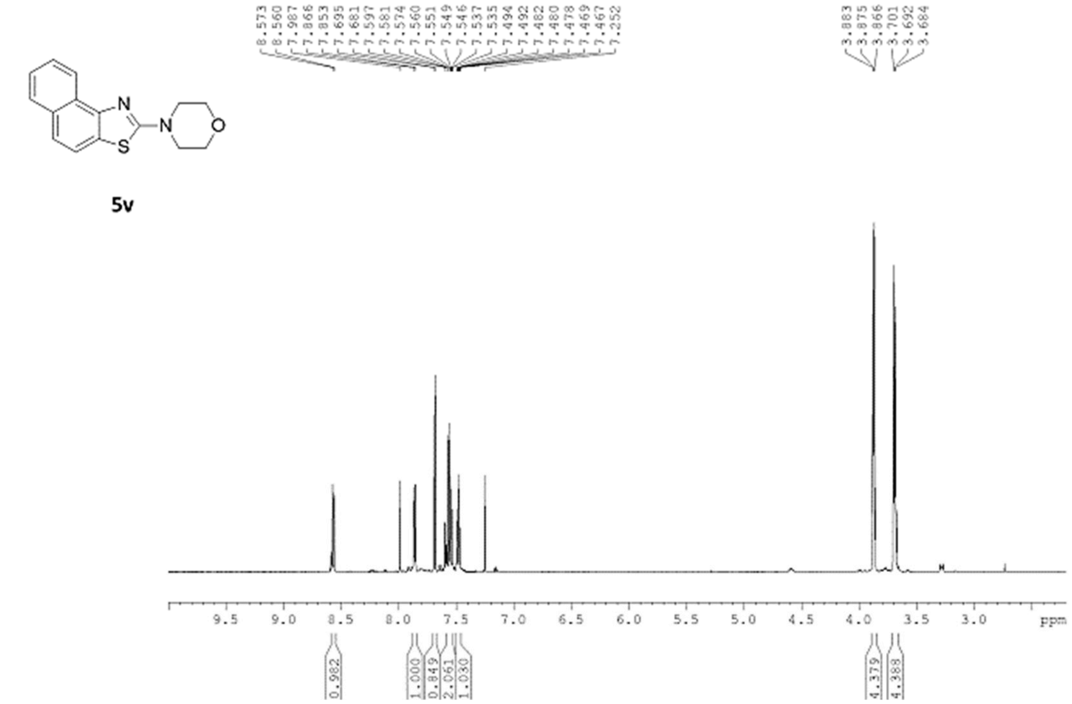

^{13}C NMR (125 MHz, $CDCl_3$) of compound **5v**

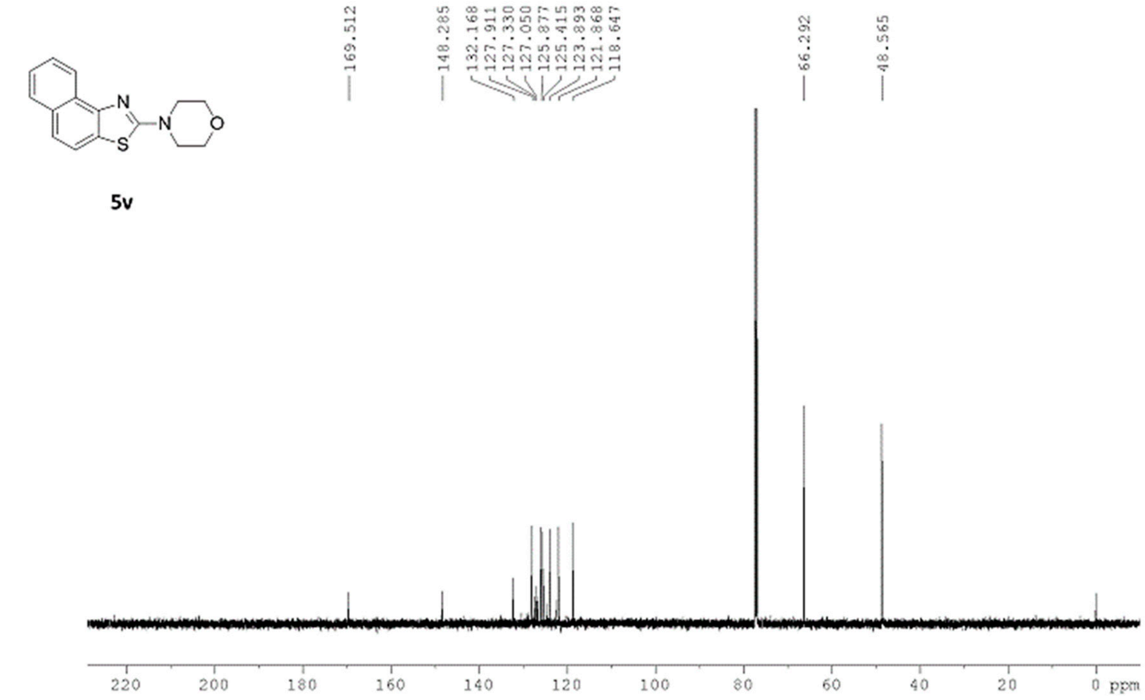

^{1}H NMR (500 MHz, $CDCl_3$) of compound **5w**

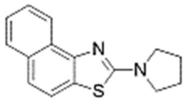

5w

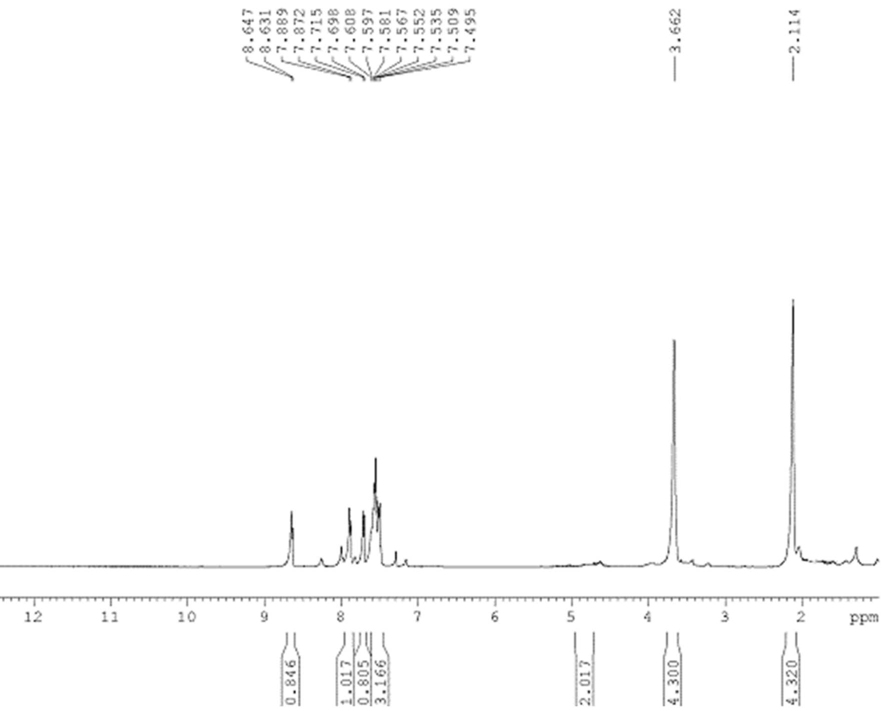

^{13}C NMR (125 MHz, $CDCl_3$) of compound **5w**

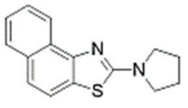

5w

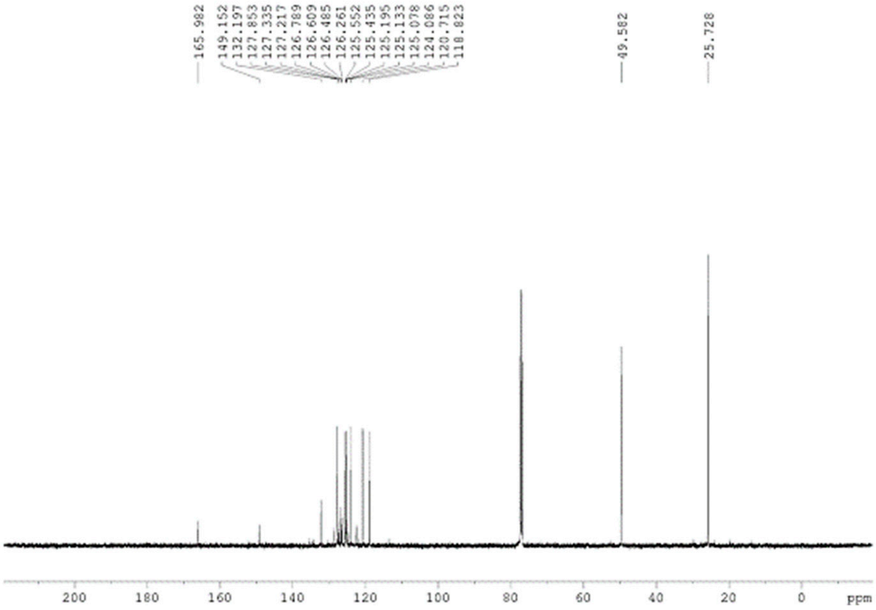

^{1}H NMR (200 MHz, $CDCl_3$) of compound **6b**

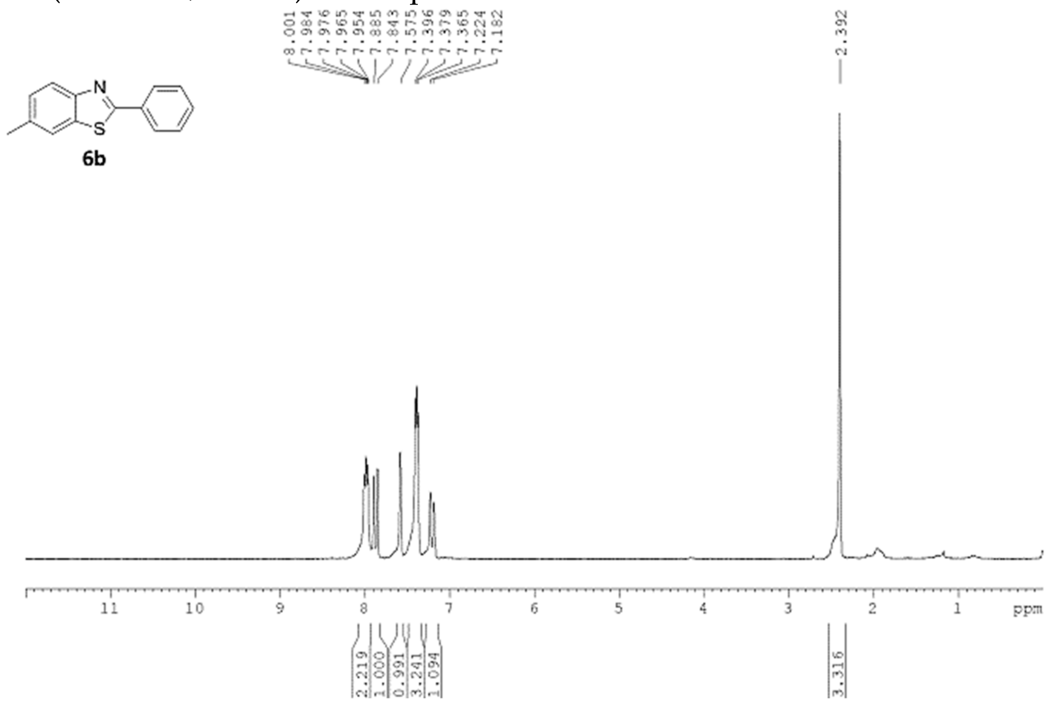

^{13}C NMR (50 MHz, $CDCl_3$) of compound **6b**

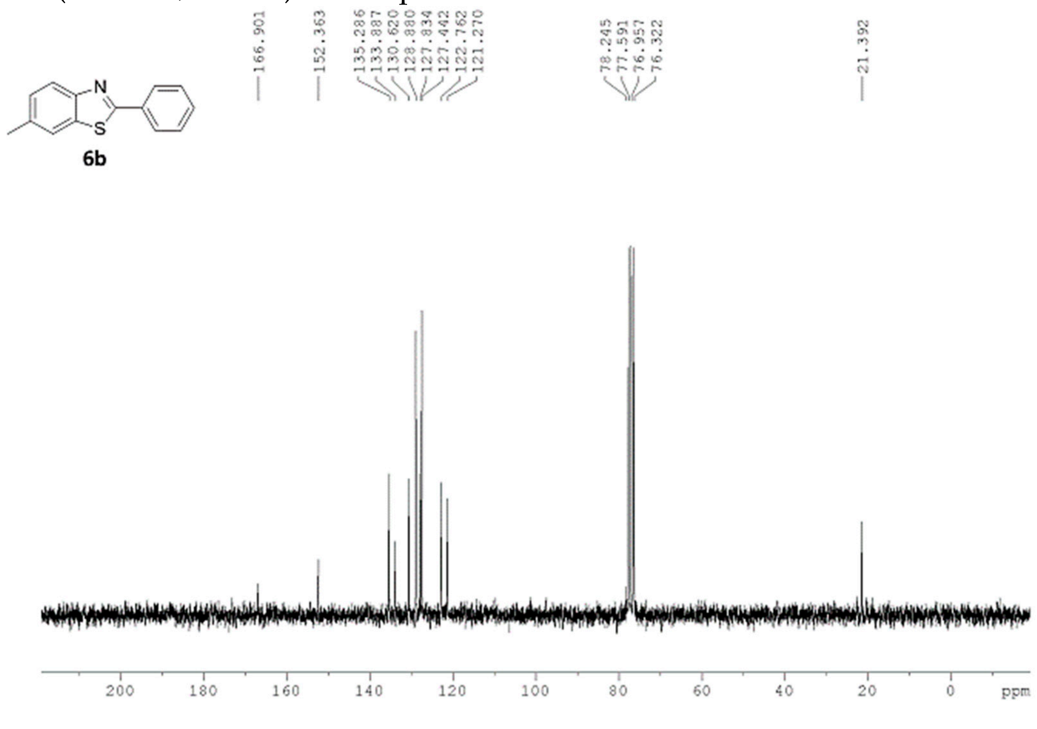

^{1}H NMR (500 MHz, $CDCl_3$) of compound **6c**

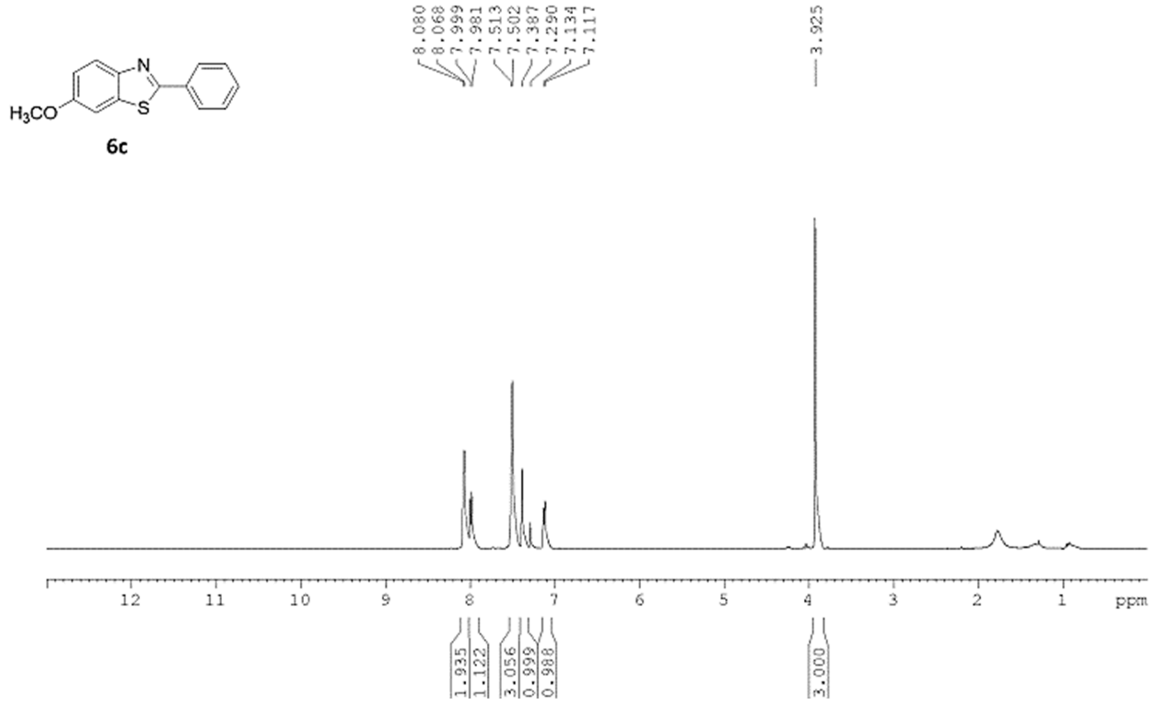

^{13}C NMR (125 MHz, $CDCl_3$) of compound **6c**

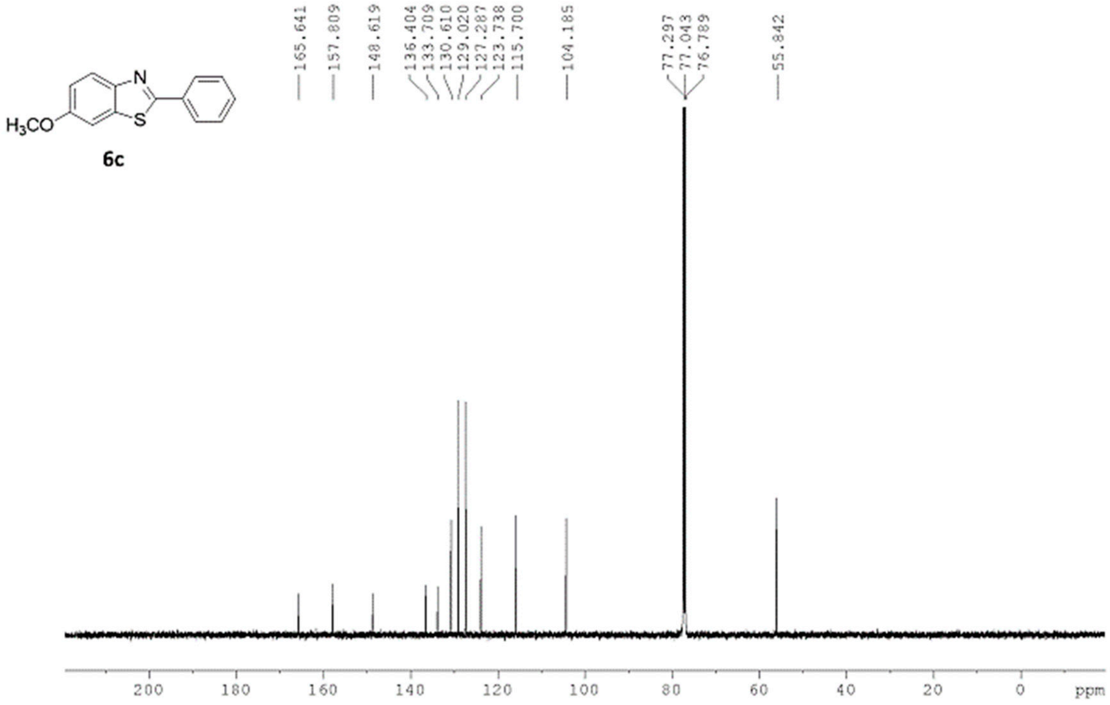

^{1}H NMR (500 MHz, $CDCl_3$) of compound **6d**

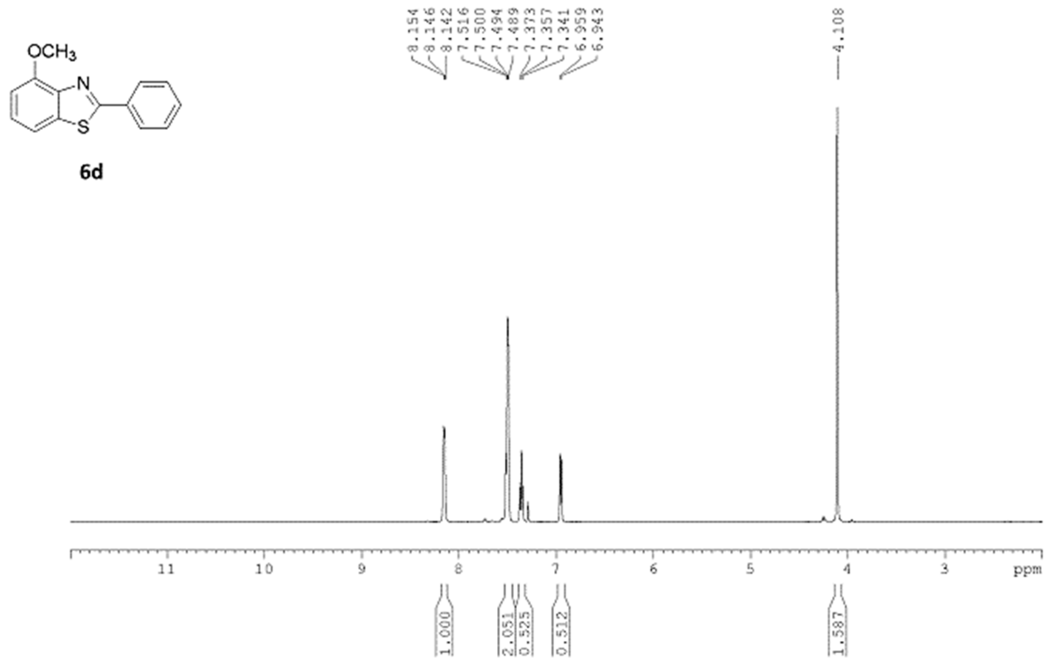

6d

^{13}C NMR, APT (125 MHz, $CDCl_3$) of compound **6d**

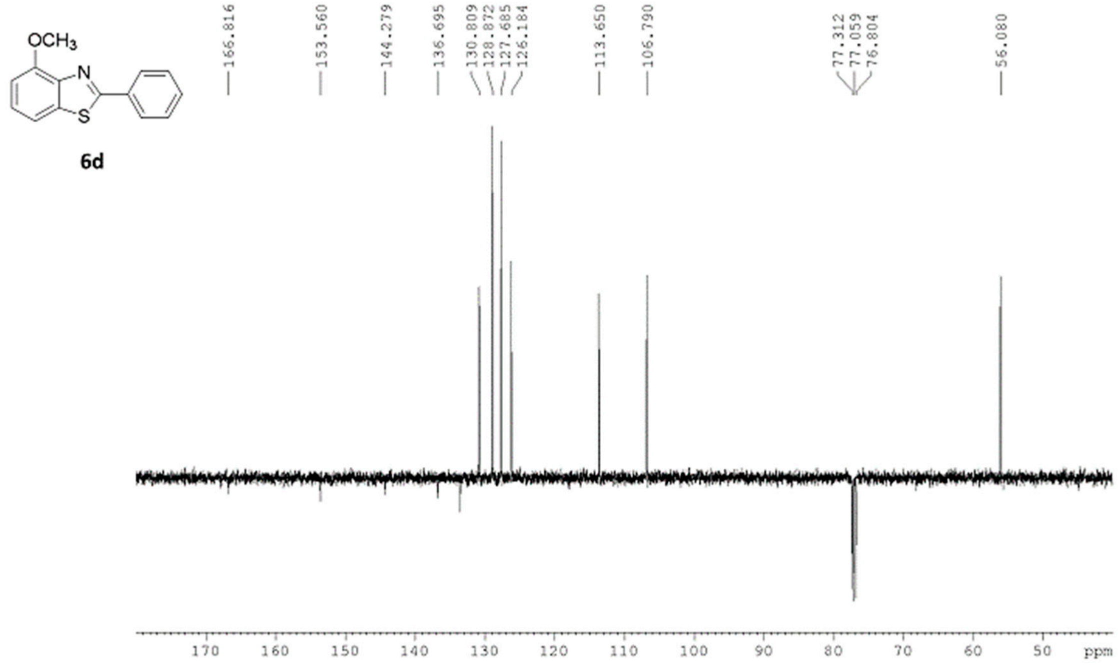

6d

^{1}H NMR (500 MHz, $CDCl_3$) of compound **6e**

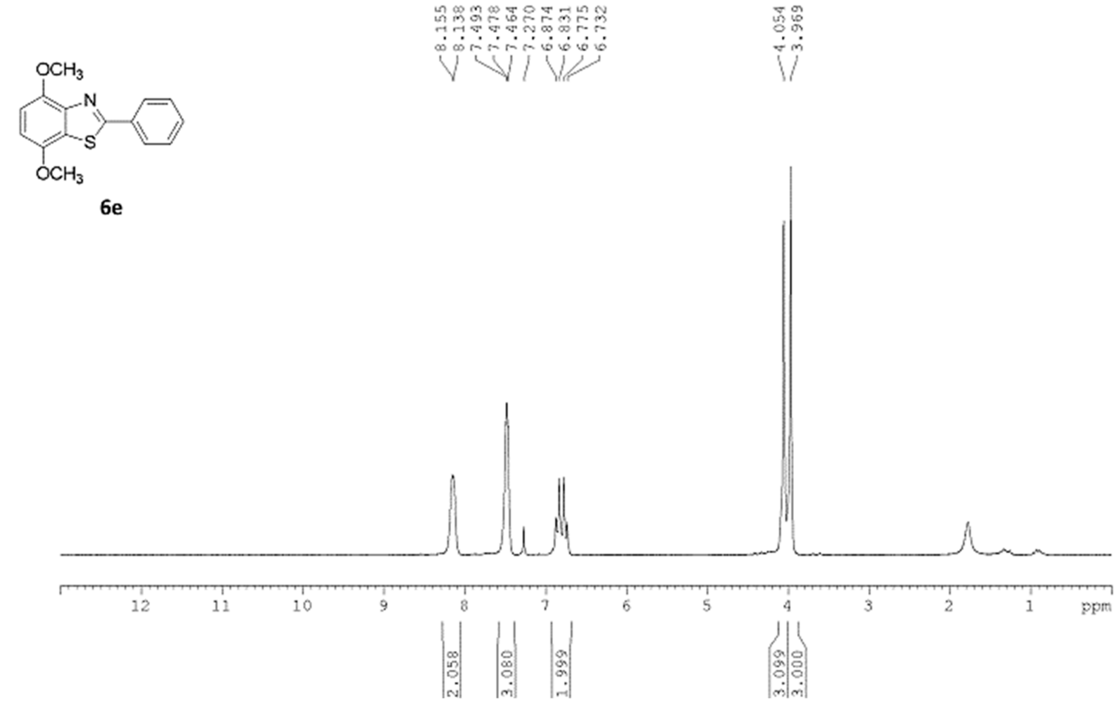

^{13}C NMR (125 MHz, $CDCl_3$) of compound **6e**

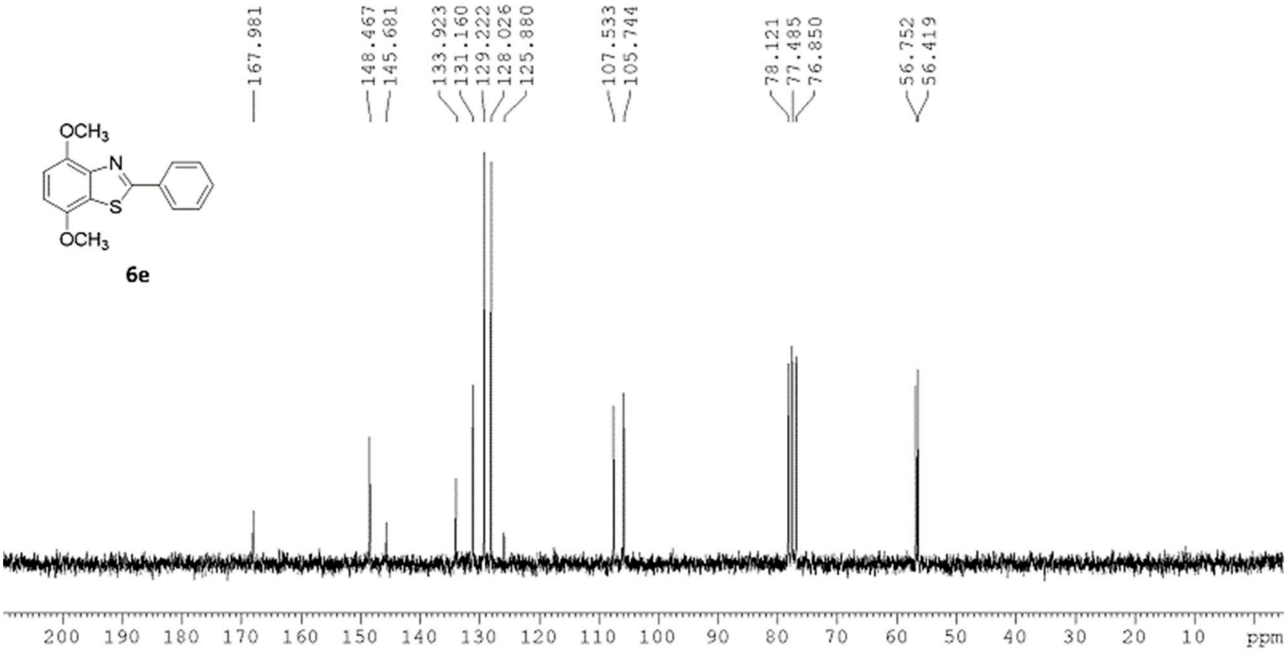

^{1}H NMR (500 MHz, $CDCl_3$) of compound **6f**

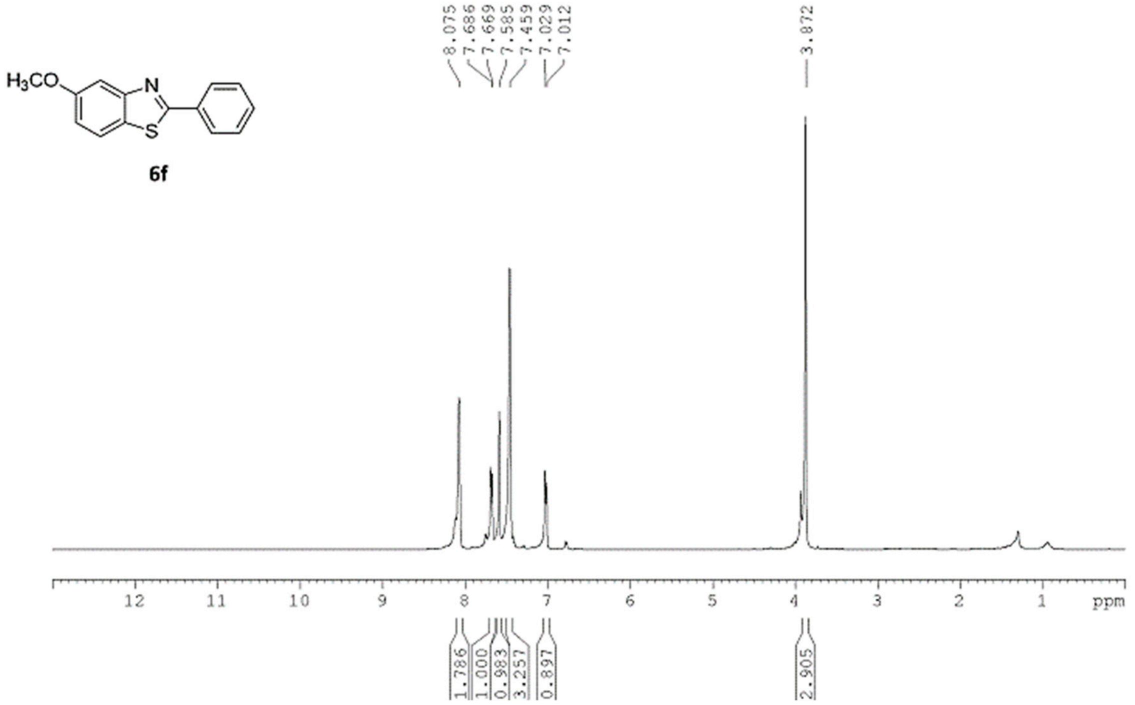

^{13}C NMR, APT (125 MHz, $CDCl_3$) of compound **6f**

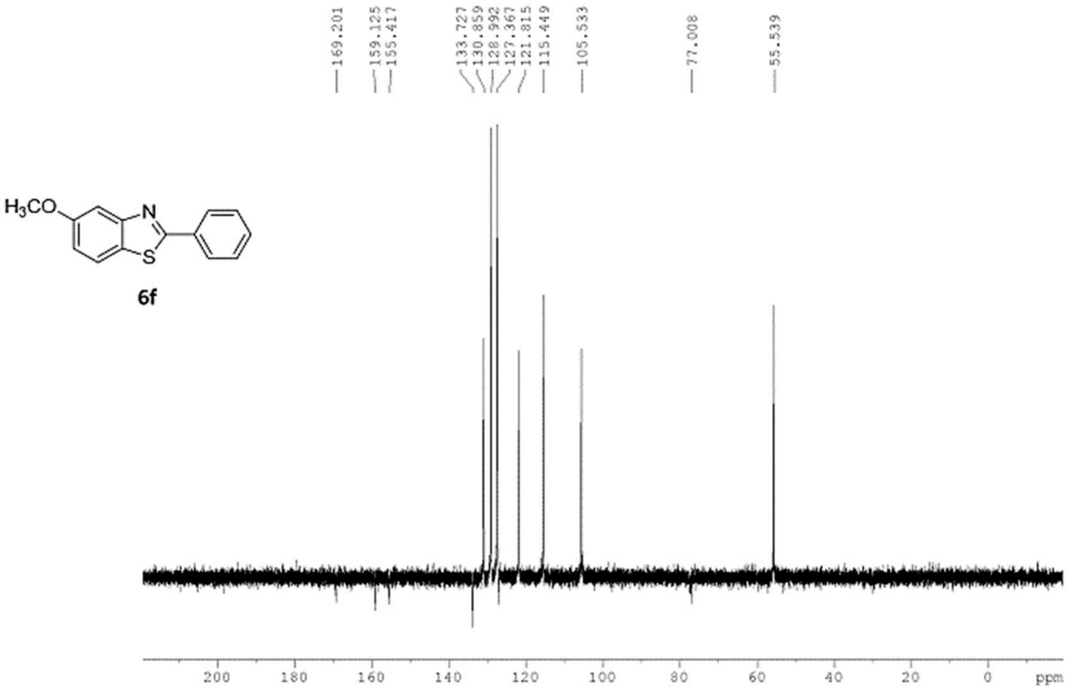

^{1}H NMR (500 MHz, $CDCl_3$) of compound **7**

7

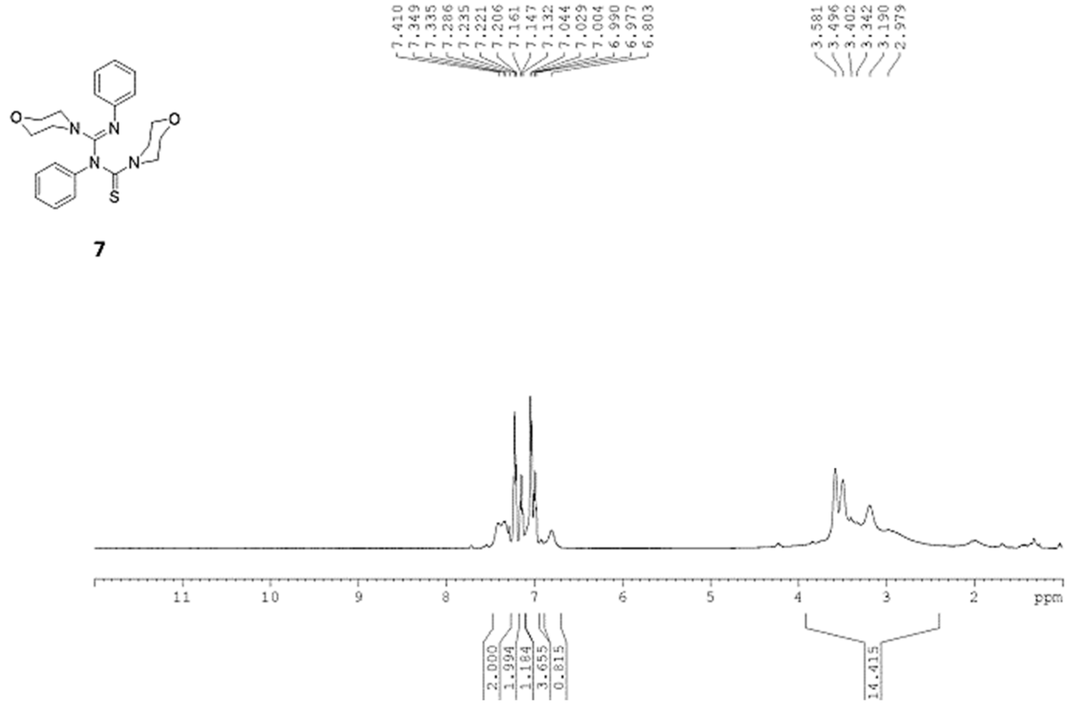

^{13}C NMR (125 MHz, $CDCl_3$) of compound **7**

7

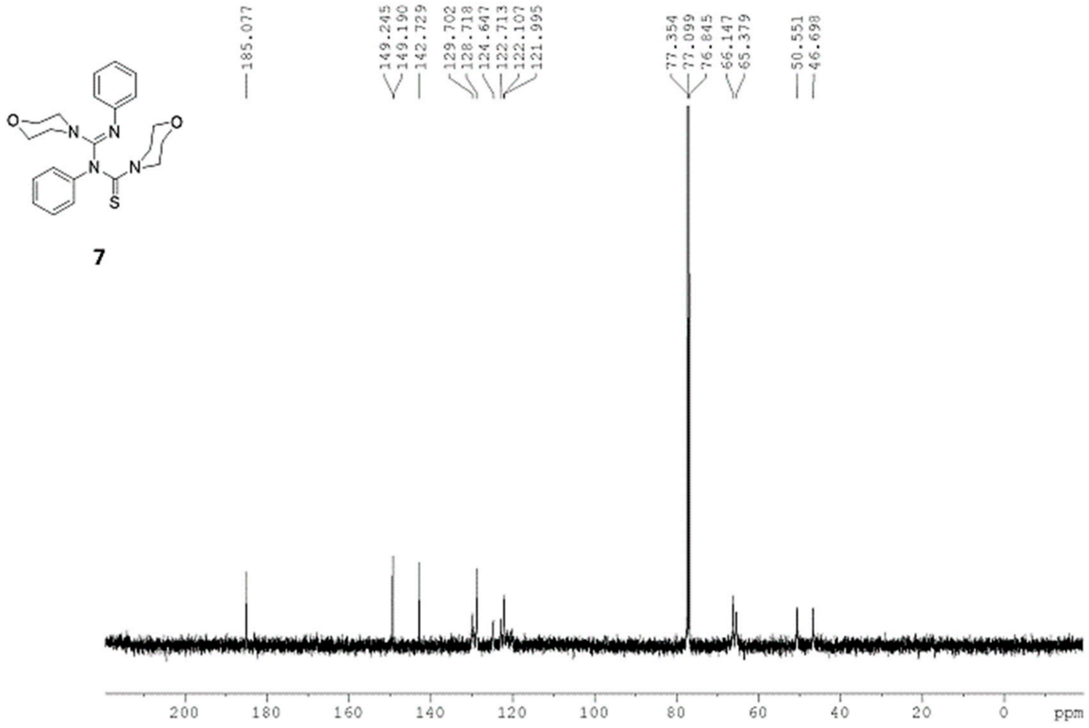

Supplement: Supplementary file 1 [file molecules-27-07876-s001.zip › molecules-1897050-supplementary.pdf]
